# Perturbation of ubiquitin homeostasis promotes macrophage oxidative defenses

**DOI:** 10.1038/s41598-019-46526-9

**Published:** 2019-07-15

**Authors:** Marie-Eve Charbonneau, Karla D. Passalacqua, Susan E. Hagen, Hollis D. Showalter, Christiane E. Wobus, Mary X. D. O’Riordan

**Affiliations:** 1 Department of Microbiology and Immunology, University of Michigan Medical School, Ann Arbor, Michigan 48109 United States of America; 2 Department of Medicinal Chemistry, College of Pharmacy, University of Michigan, Ann Arbor, Michigan 48109 United States of America

**Keywords:** Inflammation, Innate immunity

## Abstract

The innate immune system senses microbial ligands through pattern recognition and triggers downstream signaling cascades to promote inflammation and immune defense mechanisms. Emerging evidence suggests that cells also recognize alterations in host processes induced by infection as triggers. Protein ubiquitination and deubiquitination are post-translational modification processes essential for signaling and maintenance of cellular homeostasis, and infections can cause global alterations in the host ubiquitin proteome. Here we used a chemical biology approach to perturb the cellular ubiquitin proteome as a simplified model to study the impact of ubiquitin homeostasis alteration on macrophage function. Perturbation of ubiquitin homeostasis led to a rapid and transient burst of reactive oxygen species (ROS) that promoted macrophage inflammatory and anti-infective capacity. Moreover, we found that ROS production was dependent on the NOX2 phagocyte NADPH oxidase. Global alteration of the ubiquitin proteome also enhanced proinflammatory cytokine production in mice stimulated with a sub-lethal dose of LPS. Collectively, our findings suggest that major changes in the host ubiquitin landscape may be a potent signal to rapidly deploy innate immune defenses.

## Introduction

The innate immune system provides rapid protective responses to various stimuli including injury and infection. To promote inflammatory responses, immune cells initiate signaling cascades through pattern recognition receptors (PRR), which recognize diverse exogenous pathogen-associated molecular patterns (PAMP) or endogenous damage-associated molecular patterns (DAMP)^1^. The classic PRR model, however, does not explain immune discrimination between pathogenic and non-pathogenic microbes. Indeed, emerging data suggest that the innate immune system can detect conserved pathogen-induced processes such as changes in the activation state of small Rho GTPases caused by microbial infection^2–4^. The term HAMP (homeostasis-altering molecular processes) has been proposed to reflect that cells detect changes in cytoplasmic homeostasis induced during infection^5^, such as activation of the inflammasome sensor NLRP3, triggered by a wide range of signals with no structural homologies. The HAMP-sensing hypothesis is analogous to the “guard hypothesis” in plant immunity, where disruption of specific cellular interactions triggers defense responses^6^. However, the extent of cellular perturbations that mammalian cells can recognize remains to be fully defined.

A hallmark of these models is the existence of sensing mechanisms that indirectly recognize the presence of pathogens by monitoring the integrity of key host cell processes^6^. In plants, one way that homeostasis sensors can trigger downstream signaling during microbial infection is through alteration of post-translational modification (PTM), like protein phosphorylation, ubiquitination and glycosylation^7^. Protein ubiquitination is a reversible PTM that occurs by covalent attachment of an ~8.5 kDa ubiquitin molecule to a target protein^8^. Ubiquitin comprises up to 5% of total cellular protein content and is critical for regulation of many cellular processes including protein degradation, signal transduction and innate immune signaling^9,10^. Therefore, tight control of cellular ubiquitin regulation is crucial for homeostasis^11^. Although this control occurs in part through the ubiquitin-proteasome system (UPS), a number of ubiquitination events in cells are associated with non-degradative regulatory functions^12^. Control of protein ubiquitination occurs through the proteolytic action of deubiquitinases (DUBs), which remove or trim ubiquitin chains from modified proteins^13,14^. Another notable player that impacts ubiquitinated protein dynamics is the hexameric AAA ATPase p97 (also called VCP/CDC48)^15,16^. Among other functions, p97 is involved in quality control processes that result in targeting and translocation of ubiquitinated proteins for remodeling, recycling or degradation^17–19^. Of note, while p97 is a component of the UPS, its inhibition results in unique changes to the ubiquitin proteome compared to proteasome inhibition, emphasizing a distinct role for p97 in regulatory ubiquitination events^12^. In immune cells, multiple components involved in modulating the ubiquitin proteome are associated with regulation of inflammatory responses, exemplified by the activity of many DUBS, including A20, DUBA, MYSM1 and OTULIN on immune signaling^20–23^.

Given the importance of ubiquitination for many cellular processes, it is not surprising that pathogens have evolved to exploit or alter the ubiquitination system to promote infection^24^. Indeed, many pathogens encode molecular mimics of E3 ubiquitin ligases or DUBs in order to exploit the host ubiquitination machineries to their own advantage^24,25^. More importantly, recent proteomic studies reveal that infection causes major alterations in the host ubiquitination/sumoylation (small ubiquitin-related modifier) profile. *Salmonella* Typhimurium infection of human colon cells causes substantial changes in the ubiquitination landscape and intoxication of cells with the LLO pore-forming toxin from *Listeria monocytogenes* or infection with live bacteria causes global alteration of the sumoylated proteome^26–28^. These studies reinforce the idea that changes in protein ubiquitination are a major consequence of infection by pathogens. It would therefore be advantageous for host cells to detect global alterations in the ubiquitin proteome in order to activate defense mechanisms.

Here we investigate the effect of perturbing ubiquitin homeostasis, a common consequence of infection by pathogenic microbes, on the macrophage immune response. Instead of using a complex infection model where multiple host signaling pathways are altered simultaneously, we employed a targeted chemical biology approach by using small-molecule compounds to perturb the cellular ubiquitin proteome *in vitro*^29–31^. Using DUB inhibitors and a modulator of the p97 ATPase, we show that macrophages respond to acute perturbation of cellular ubiquitin homeostasis by generating a robust and transient NOX2-dependent burst of reactive oxygen species (ROS) that promotes macrophage anti-infective capacity. Our results are consistent with a model whereby perturbation of the host ubiquitin landscape can be detected by immune cells as a signal for activation of the macrophage anti-microbial and inflammatory arsenal.

## Results

### Molecules altering the ubiquitin proteome induce accumulation of ubiquitinated proteins in macrophages

**Figure 1:**
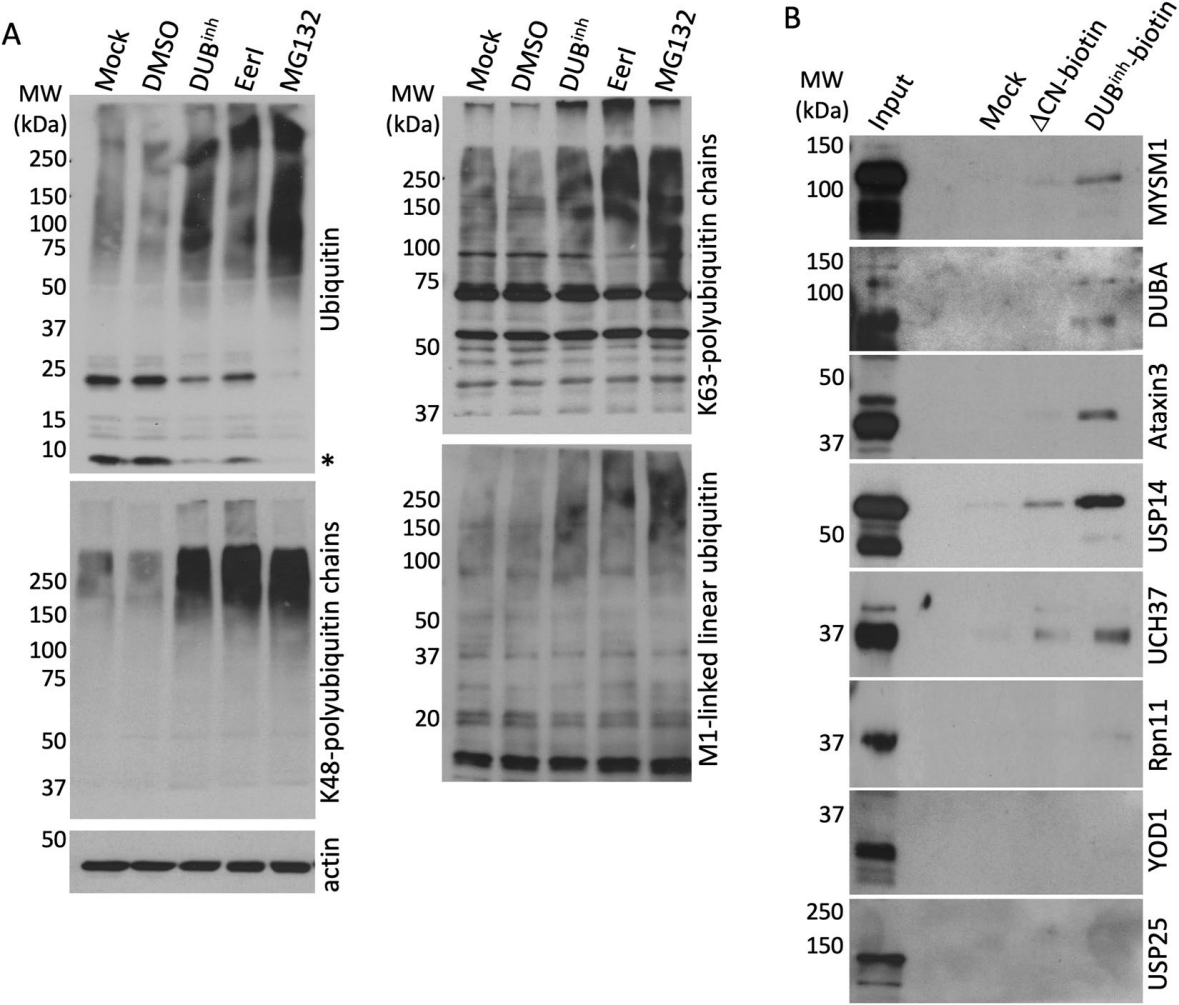
Perturbation of the ubiquitin proteome in macrophages results in accumulation of high-molecular weight ubiquitinated proteins. (**A**) Immunoblots of RAW264.7 whole cell lysates following treatment with DUB^inh^ (3.5 µM, 1 h), EerI (10 µM, 1 h) or MG132 (5 µM, 2 h) probed with specific antibodies for total mono- and poly-ubiquitinated proteins, K63-, K48- or linear M1-linked specific polyubiquitin chains and β-actin. The asterisk represents the free monoubiquitin. (**B**) RAW264.7 whole cell lysates were incubated with DUB^inh^-Biotin or ΔCN-biotin before immunoprecipitation using streptavidin-coated beads. The retained proteins were immunoblotted with antibodies for MYSM1 (95 kDa), DUBA (61 kDa), Ataxin3 (42 kDa), USP14 (60 kDa), UCH37 (37 kDa), Rpn11 (35 kDa), YOD1 (37 kDa) and USP25 (126 kDa). Data are representative of three independent experiments. Full-length immunoblots are presented in Supplementary Figs S6 and S7.

To globally perturb ubiquitin homeostasis in macrophages, we used a chemical biology approach. We previously characterized two structurally related DUB inhibitors (DUB^inh^), G9 and C6 (Supplementary Fig. S1A), that cause global alteration of protein ubiquitination in macrophages at concentrations that preserve cell viability^29,30^. To better understand the impact of DUB^inh^ treatment on macrophage ubiquitination status, we compared the effect of DUB^inh^ with two other small molecules known to disrupt cellular ubiquitin homeostasis. RAW264.7 cells, a murine macrophage-like cell line, were treated for 1 h with DUB^inh^, 1 h with the p97/VCP modulator Eeyarestatin I (EerI)^31^ or 2 h with the proteasome inhibitor MG132^32^. We observed that treatment of macrophages with DUB^inh^, EerI or MG132 resulted in notable increases in polyubiquitinated proteins with corresponding decreases in free ubiquitin (Fig. 1A). Ubiquitin has seven lysine residues and an amino-terminal methionine that can be linked to other ubiquitin molecules. K48-ubiquitin chains target proteins for proteasomal degradation whereas K63- and M1-linkages are often associated with regulatory signaling processes, including immune signaling^8,33^. All treatments induced an increase of K48-, K63-, and M1-specific polyubiquitin linkages (Fig. 1A). These data demonstrate that we can efficiently perturb cellular ubiquitin homeostasis in macrophages using a chemical biology approach.

DUB^inh^ treatment causes a global alteration of the cellular ubiquitination profile, suggesting that this small molecule might target many cellular DUBs. Therefore, we used a biotinylated version of this compound (DUB^inh^-biotin) as an affinity reagent to identify DUBs that interact with DUB^inh^. As a control, we used the ΔCN-biotin molecule, which lacks a cyano group that contributes to DUB^inh^ activity on target DUBs (Supplementary Fig. S1B)^29^. RAW264.7 cell lysates were incubated with DUB^inh^-biotin or ΔCN-biotin, and protein complexes were isolated with streptavidin-agarose beads. Silver staining revealed that multiple proteins of various sizes specifically bind to DUB^inh^-biotin (Supplementary Fig. S1C). To further identify binding partners, we directly analyzed by immunoblot select DUBs involved in various cellular functions, including protein degradation (USP14, UCH37, Rpn11)^34–36^, endoplasmic reticulum-associated degradation (ERAD) (YOD1, Ataxin-3, USP25)^37–39^ and innate immune signaling (MYSM1, DUBA, USP14)^21,22,40^. Six out of the eight interrogated DUBs, MYSM1, DUBA, Ataxin-3, UCH37, USP14 and to a lesser extent Rpn11, were preferentially associated with DUB^inh^-biotin compared to ΔCN-biotin (Fig. 1B). Our results are in agreement with a previous report suggesting that many previously described DUB inhibitors display activity against a wide range of DUBs^41^. Our results therefore supported the use of DUB^inh^ as a good chemical tool to assess the effects of broad perturbation of ubiquitin homeostasis on the macrophage innate immune response.

### DUB inhibition induces ROS production in macrophages

**Figure 2:**
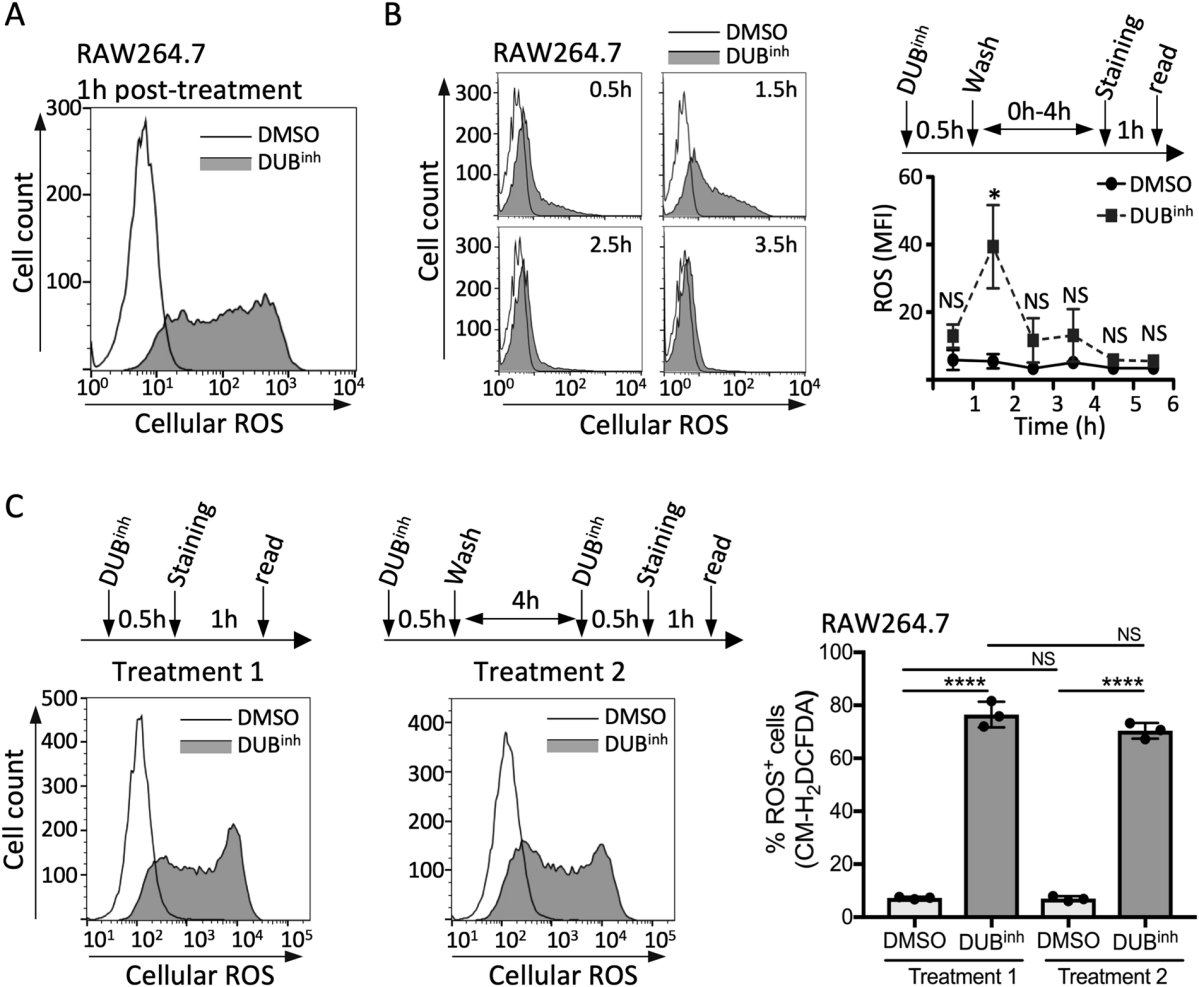
Inhibition of cellular DUBs in macrophages induces transient ROS generation. (**A**) FACS analysis of RAW264.7 cells treated with DUB^inh^ for 0.5 h before staining with 5 µM CM-H_2_DCFDA. (**B**) Time course of ROS production in RAW264.7 following treatment with 3.5 µM DUB^inh^. The strategy used for the time course is depicted and representative plots are shown for each time point. (**C**) RAW264.7 cells were treated with DUB^inh^ for 0.5 h before staining for ROS production as described above (treatment 1). Alternatively, treated macrophages were allowed to recovery for 4 h in media before a second challenge with 3.5 µM DUB^inh^. Cells were subsequently stained for ROS production (treatment 2). The left panels show representative histograms whereas the right panel shows the percentage of cells stained for ROS (% ROS^+^ cells) obtained from three independent experiments. The mean fluorescence intensity (MFI) and the percentage of ROS+ cells were calculated using FlowJo software. Significant differences were calculated using two-tailed Student’s t test or one-way ANOVA and Tukey’s multiple comparison test on the unmodified data (NS, not significant, *p < 0.05, ****p < 0.0001).

Macrophages generate reactive oxygen species (ROS) as a potent effector mechanism to kill invading pathogens and promote immune signaling. To determine whether DUB^inh^ treatment affects ROS production, we used the redox-sensitive fluorescent indicator chloromethyl-2′,7′-dichlorodihydrofluorescein diacetate (CM-H_2_DCFDA). We observed by live cell flow cytometry analysis that ROS production was increased in RAW264.7 cells treated with DUB^inh^ compared to DMSO-treated cells (Fig. 2A). Similar results were obtained using the DUB^inh^ C6 (Supplementary Fig. S2A). Co-treatment of RAW264.7 cells with DUB^inh^ and the ROS scavenger *N*-acetylcysteine (NAC) or with reduced L-glutathione (GSH) abolished ROS production (Supplementary Fig. S2B). DUB^inh^ treatment also induced a burst of ROS early after treatment in THP-1 and U937 cells, two human monocyte cell lines, suggesting this response is conserved in both mouse and human cells of the macrophage/monocyte lineage (Supplementary Fig. S2C,D). To gain insight into the kinetics of ROS production in response to DUB inhibition, we measured ROS generation at different times after treatment (Fig. 2B). The increase in ROS production was observed as early as 0.5 h post-treatment with a peak response 1.5 h after addition of DUB^inh^. Strikingly, ROS levels decreased as early as 2.5 h post-treatment and returned to a level comparable to DMSO-treated macrophages by 4.5 h, suggesting that perturbation of ubiquitin homeostasis induces a transient increase in ROS generation. We also assessed the potential for macrophages to respond to a second challenge with the DUB inhibitor. To this end, macrophages were treated with the DUB inhibitor for 0.5 h and allowed to recover for 4 h before challenging them a second time. As shown in Fig. 2C, cells responded to a second challenge by producing a similar burst of ROS, demonstrating that the trigger signal for ROS generation in macrophages is transient and can be effectively re-set.

**Figure 3:**
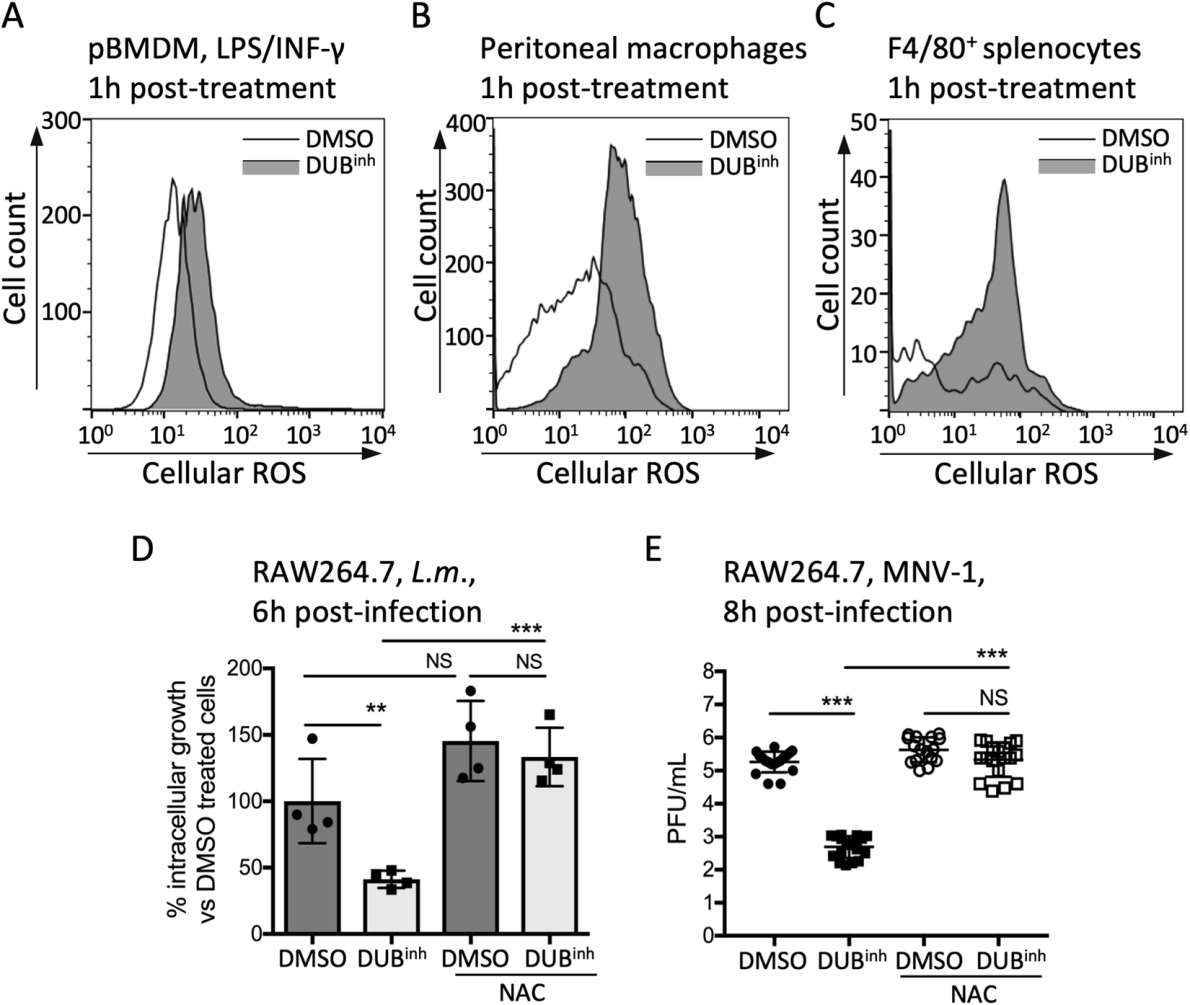
DUB^inh^ promotes a ROS production that enhance macrophages antimicrobial effector mechanisms. (**A**) pBMDM incubated overnight with 100 ng/ml of LPS and INF-γ were treated for 0.5 h with 3.5 µM DUB^inh^ before staining with ROS dye. Peritoneal macrophages (**B**) and splenocytes (**C**) were treated for 0.5 h with 1 µM DUB^inh^ before staining with 1.25 µM and 2 µM CM-H_2_DCFDA, respectively. Subsequent staining of splenocytes with F4/80^+^ antibody was performed to label macrophages. (**D**) RAW264.7 cells were incubated with 10 mM NAC or medium only for 0.5 h. DUB^inh^ was added on top at a final concentration of 3.5 µM for 0.5 h. Cells were infected with *L. monocytogenes* (MOI 1) for 0.5 h, washed and incubated for 6 h in medium containing 10 µg/ml of gentamicin before enumeration of intracellular bacteria. The data represent percent of intracellular *L. monocytogenes* growth compared to DMSO-only treated cells. (**E**) RAW264.7 cells were pre-treated with 10 mM NAC or medium in combination with 2.5 µM DUB^inh^ C6 or equivalent volume of DMSO for 0.5 h before infection with MNV-1 (MOI 5) for 1 h on ice. Viral titers were determined from lysates harvested at 8 h p.i. All results are from at least three independent experiments. Significant differences were calculated using one-way ANOVA and Tukey’s multiple comparison test on the unmodified data (NS, not significant, **p < 0.01, ***p < 0.001).

We next assessed the relevance of our findings in primary immune cells and observed that DUB inhibition caused a burst of ROS in primary bone-marrow derived macrophages (pBMDM) (Fig. 3A). Similarly, treatment of peritoneal-resident macrophages or splenic macrophages with DUB^inh^ resulted in a significant increase in ROS generation (Fig. 3B,C). These results show that ROS generation following perturbation of ubiquitin homeostasis is a functional response in primary macrophages. ROS produced by phagocytes can have antimicrobial activity against a broad range of pathogens. We previously reported that DUB inhibition in RAW264.7 cells resulted in lower pathogen burden using *Listeria monocytogenes* and murine norovirus (MNV-1), a surrogate for human norovirus, as model intracellular pathogens^29,30^. To explore the relevance of ROS generation during DUB inhibition, we examined the effect of the ROS scavenger NAC and the anti-oxidant GSH on the antimicrobial activity of DUB^inh^ compounds. As shown before^29^, treatment of RAW264.7 cells with DUB^inh^ significantly reduced the number of *L. monocytogenes* intracellular colony forming units (CFU) at 6 h p.i. (Fig. 3D). However, addition of NAC or treatment with GSH during DUB inhibition significantly reduced the antimicrobial effect of DUB^inh^ against *L. monocytogenes*, consistent with a role for ROS generation as an antimicrobial effector (Figs 3D and S2E). Likewise, addition of NAC or GSH abolished the effect of DUB inhibition on MNV-1 infection (Figs 3E and S2F). Collectively, our results suggest that perturbation of ubiquitin homeostasis using DUB^inh^ in macrophages promotes a transient and robust ROS burst that can effectively enhance antimicrobial effector functions.

### Targeting function of the ubiquitin-dependent segregase p97 in macrophages induces ROS production

**Figure 4:**
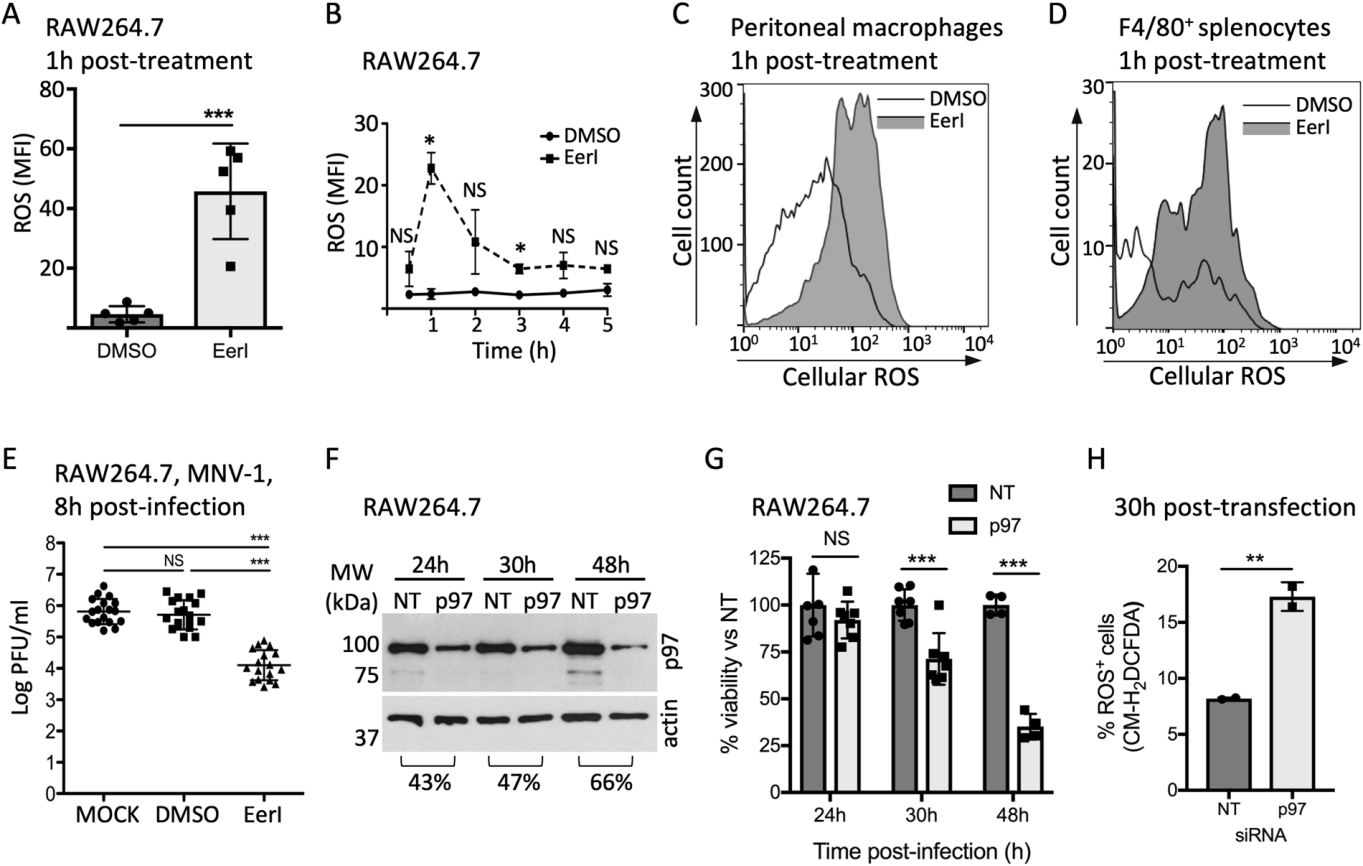
Perturbation of p97 activity in macrophages induces ROS generation. (**A**) RAW264.7 cells were treated with 10 µM EerI for 1 h before staining with 5 µM CM-H_2_DCFDA. (**B)** Time course of ROS production in RAW264.7 following treatment with 10 µM EerI for 1 h. The MFI was calculated using FlowJo software. Peritoneal macrophages (**C**) and splenocytes **(D**) were treated for 1 h with 10 µM EerI before staining with 1.25 µM and 2 µM CM-H_2_DCFDA, respectively. Subsequent staining of splenocytes with F4/80^+^ antibody was performed to label macrophages. All FACS plots are representative of three independent experiments. (**E**) RAW264.7 cells were treated with 10 µM EerI for 1 h before infection with MNV-1 (MOI 5) for 1 h on ice. Viral titers were determined by counting plaque-forming units (PFU) from lysates harvested at 8 h p.i. (**F**) Whole cells lysates of RAW264.7 transfected with siRNA against p97 or a non-targeted control were analyzed by immunoblotting against p97. Full-length blots are presented in Supplementary Fig. 8C. Cell viability was determined using the WST-1 reagent and results represent the percent viability compared to NT control transfected cells. (**H**) At 30 h post-transfection, cells were stained with 5 µM CM-H_2_DCFDA for 0.5 h. The percentage of cells stained for ROS (% ROS^+^ cells) was obtained from two independent experiments. Significant differences were calculated using two-tailed Student’s t test or one-way ANOVA and Tukey’s multiple comparison test on the unmodified data (NS, not significant, *p < 0.05, **p < 0.01, ***p < 0.001). Full-length immunoblots are shown in Supplementary Fig. S7.

If perturbation of ubiquitin homeostasis is a global signal that promotes ROS production in macrophages, this response should also be induced by alteration of other regulators of the ubiquitin proteome. P97 is a gatekeeper for quality control of many ubiquitin-dependent cellular processes, including ER-, mitochondria- and chromatin-associated degradation, ribosome-associated quality control, cytosolic degradation, membrane trafficking and macroautophagy^16,42^. Consistent with previous reports, we observed that alteration of p97 function using the small molecule EerI caused accumulation of ubiquitinated proteins in macrophages at a concentration that preserves cell viability (Fig. 1A and S3A), and therefore EerI represented a method for altering the ubiquitin proteome distinct from DUB^inh^ treatment^31^. Of note, EerI was shown to directly bind p97, but rather than inhibiting ATPase activity, EerI treatment altered p97 interaction with co-factors, which are essential to support p97 function^31^. Treatment of RAW264.7 cells for 1 h with EerI resulted in a robust and transient increase in ROS production in macrophages (Figs 4A,B and S3B). EerI treatment also induced ROS production in the human monocyte cells lines THP-1 and U937 (Supplementary Fig. S3C,D), and in primary resident-peritoneal or splenic macrophages, suggesting a physiologically relevant response (Fig. 4C,D). As a second way to disrupt p97 function, we treated cells with DBeQ, a potent ATP-competitive p97 inhibitor^43^. However, we observed that DBeQ caused high toxicity at effective inhibitory concentrations, preventing further use of this compound (Supplementary Fig. S3A). To explore the potential effector function of ROS generated through EerI treatment, we used the MNV-1 infection model, as EerI inhibits *L. monocytogenes* axenic growth. Treatment of RAW264.7 cells with 5 µM EerI resulted in a ~2 log decrease in viral titer at 8 h p.i. (Fig. 4E). Therefore, disrupting p97 function also promoted ROS generation and anti-viral activity, consistent with the idea that perturbation of ubiquitin homeostasis can be sensed by macrophages and results in activation of defense mechanisms.

We also sought to further test our hypothesis using a genetic approach to target p97, using a pool of small interfering RNAs (siRNA) to deplete p97 in murine macrophages. We observed a ~65% reduction of protein level by 48 h post-transfection, however, notable loss of cell viability was also observed (Fig. 4F,G), suggesting that p97 function is crucial for long-term macrophage survival. This result is consistent with the observation that treatment with the ATP-competitive p97 inhibitor DBeQ is highly toxic for macrophages (Supplementary Fig. S3A). At 30 h post-transfection, where we obtained suitable reduction in protein levels and limited loss of cell viability, we observed a 2-fold increase in the percentage of ROS-producing live macrophages when transfected with p97 siRNA compared to NT control (Figs 4H and S3E,F). These results suggest that partial disruption of p97 function might potentiate the antimicrobial capacity of macrophages through generation of ROS, consistent with our hypothesis that global ubiquitin perturbation signals activation of innate immune defense in macrophages.

### ROS production upon perturbation of ubiquitin homeostasis in macrophages is independent of UPR activation

We showed above that a consequence of DUB inhibition was the build-up of ubiquitinated proteins, a condition that could potentially lead to ER stress and activation of the unfolded protein response (UPR), which is mediated by three resident ER sensors, IRE1, PERK and ATF6. It was also proposed that the ER can act as a surveillance platform to detect pathogen invasion and subsequently activate immune responses^44,45^. Therefore, we probed the possible role of UPR signaling in ROS generation upon perturbation of ubiquitin homeostasis. IRE1 activation was determined by measuring *Xbp*1 transcript splicing, a direct target of the endonuclease domain of IRE1, while PERK activation was assessed by looking at induction of CHOP, encoded by a gene upregulated in a PERK-dependent manner^46,47^. Thapsigargin and/or tunicamycin treatment, two known inducers of ER stress, induced CHOP expression and *Xbp1* splicing, whereas no activation of either pathway was detected in RAW264.7 cells treated with DUB^inh^ (Supplementary Fig. S4A,B). To test whether UPR sensors were involved in generation of antimicrobial ROS upon DUB inhibition, we used the IRE1 inhibitor 4µ8c^48^ and the PERK inhibitor GSK 2606414 (GSK-PERK)^49^ and showed that these drugs did not rescue *L. monocytogenes* growth reduction in RAW264.7 cells upon DUB inhibition (Supplementary Fig. S4C,D). To directly test the potential role of UPR signaling in DUB^inh^-mediated ROS production, we treated cells with tauroursodeoxycholic acid (TUDCA), a chemical chaperone that ameliorates ER stress^50^. RAW264.7 cells produced similar levels of ROS after DUB^inh^ treatment in the presence or absence of TUDCA (Supplementary Fig. S4E), suggesting that ROS generation following ubiquitin homeostasis perturbation by DUB^inh^ does not rely on the unfolded protein response.

### Alteration of proteasome function is not sufficient to induce ROS generation in macrophages

**Figure 5:**
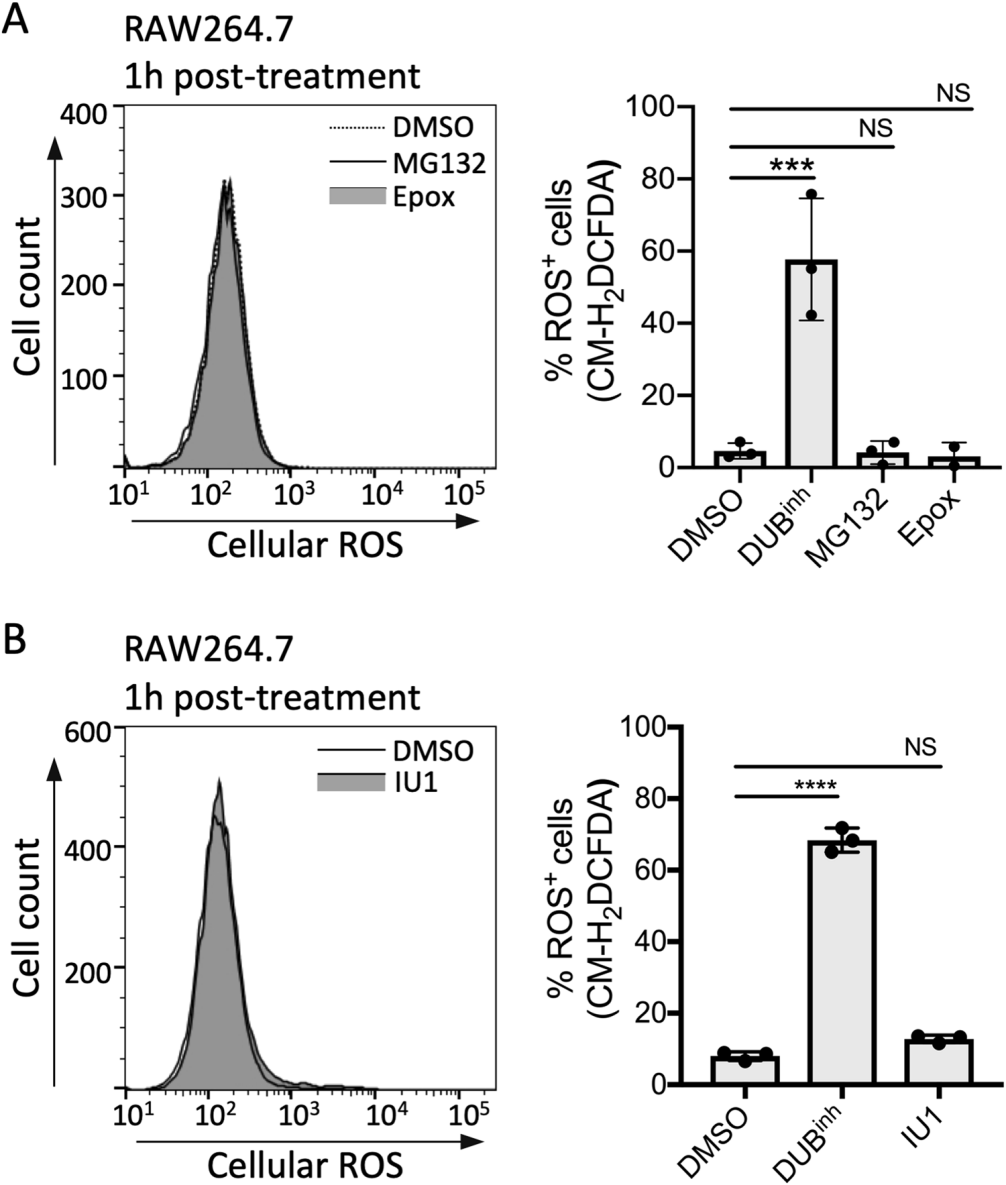
Inhibition of proteasome function is not sufficient to induce ROS generation in macrophages. (**A**) RAW264.7 cells were treated with 5 µM MG132 or 0.5 µM epoxomicin for 1 h before staining for ROS. (**B**) RAW264.7 cells were treated for 1 h with 100 µM IU1, a USP14-specific inhibitor, before staining for ROS. FACS plots are representative of 3 independent experiments. The left panels show representative histograms where right panels show the percentage of cells stained for ROS (% ROS^+^ cells) obtained from 3 independent experiments. Significant differences were calculated using one-way ANOVA and Tukey’s multiple comparison test on the unmodified data (NS, not significant, ***p < 0.001, ****p < 0.0001).

DUB function is integral to the ubiquitin-proteasome system, and we showed above that inhibition of proteasome function also resulted in global perturbation of cellular ubiquitination (Fig. 1A). Moreover, we showed that three DUBs associated with the proteasome, USP14, UCH37 and to a lesser extent Rpn11, were preferentially precipitated with our DUB^inh^-biotin compound (Fig. 1B). Consequently, we next investigated the effect of proteasome inhibition or specific inhibition of the DUB USP14 on ROS production by macrophages. RAW264.7 cells were treated for 1 h with MG132 or epoxomicin (Fig. 5A), two commonly used proteasome inhibitors, or with the USP14 inhibitor IU1^51^ (Fig. 5B) and ROS production was measured as before. In contrast to the results obtained with the DUB^inh^ and EerI, no increase in ROS generation was observed with any of the proteasome inhibitors tested. It is important to note that EerI and other well-studied DUB inhibitors have been shown to have no direct effect on proteasome proteolytic activity^52^. Also, p97 inhibition resulted in distinct alterations of the ubiquitin-modified proteome compared to proteasome inhibition, despite their similar effect on global ubiquitin homeostasis^12^. These observations reveal that different insults to the cellular ubiquitin proteome may stimulate distinct cellular responses. Accordingly, our findings raise the possibility that perturbation of ubiquitin homeostasis in macrophages by altering DUB function or p97 activity triggers defenses through ubiquitin-sensitive regulatory signals rather than through inhibition of proteasome function.

### ROS production induced by perturbation of ubiquitin homeostasis requires NOX2

**Figure 6:**
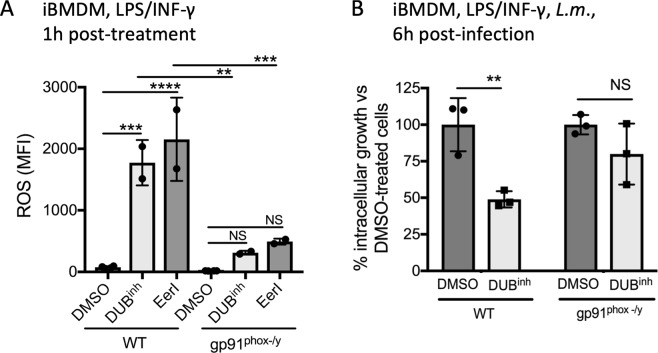
Perturbation of ubiquitin homeostasis in macrophages induces NADPH phagocyte oxidase-dependent ROS generation**. (A**) WT and *gp91*^*phox−/y*^ iBMDM were treated for 0.5 h with 3.5 µM DUB^inh^ or 10 µM EerI before staining for 0.5 h with 5 µM CM-H_2_DCFDA. Quantification of MFI from two independent experiments was calculated using FlowJo software. (**B**) WT and *gp91*^*phox−/y*^ iBMDM were treated for 0.5 h with 3.5 µM DUB^inh^ before infection with *L. monocytogenes* (MOI 1) for 0.5 h. Intracellular bacteria were enumerated at 6 h p.i. Data are from 3 independent experiments and, significant differences were calculated using one-way ANOVA with Tukey’s multiple comparison test (NS, not significant, **p < 0.01, ***p < 0.001, ****p < 0.0001).

The NADPH phagocyte oxidase complex (NOX2) is a major source of ROS in macrophages^53^. The NOX2 complex core is composed of membrane-bound p22^phox^ and gp91^phox^, the cytochrome b558 unit. Multiple cytosolic subunits are required for optimal NOX2 activation, including p40^phox^, p47^phox^, p67^phox^ and the Rac GTPase^53^. The function and localization of some of these subunits are controlled by a variety of PTMs. Moreover, other signals, including calcium flux or lipids, especially arachidonic acid, can stimulate NOX2 activation and ROS generation emphasizing the complexity of NOX2 regulation. To investigate the role of this complex in DUB^inh^-mediated ROS production, we use iBMDM isolated from *gp91*^*phox−/y*^ mice, a commonly used model to study the function of NOX2 as these mice lack a functional NOX2 complex^54^. A ~6-fold and ~4-fold decrease in ROS production in response to DUB^inh^ and EerI treatment respectively was observed in *gp91*^*phox−/y*^ iBMDM compared to WT cells (Fig. 6A). Whole cell extracts from RAW264.7 cells or iBMDM showed no consistent change in gp91^phox^ expression levels following inhibition of DUBs, eliminating the possibility that the increased ROS production observed was a direct consequence of higher gp91^phox^ protein levels (Supplementary Fig. S5A). Moreover, analysis of whole cell extracts of iBMDM showed no difference for the expression level of p22^phox^ and p67^phox^, two other required components of NOX2 complex (Fig. S5B). As macrophages possess other NADPH oxidase enzymes, we tested the effect of NOX1 or NOX4 loss of function on ROS generation after DUB^inh^ treatment using primary BMDM isolated from *Nox1*^−/−^ or *Nox4*^−/−^ mice and observed that these two enzymes are dispensable for ROS generation (Supplementary Fig. S5C). If NOX2 is a major effector mechanism activated by cellular sensing of altered ubiquitin homeostasis, we would expect the anti-infective activity of DUB^inh^ compound to be reduced in cells lacking this NADPH oxidase complex. Accordingly, DUB inhibition in WT iBMDM resulted in a significant decrease of *L. monocytogenes* growth at 6 h p.i, whereas no significant difference was observed for *L. monocytogenes* grown in *gp91*^*phox−/y*^ iBMDM (Fig. 6B). Taken together, our data suggest that upon perturbation of ubiquitin homeostasis, macrophages generate a burst of ROS that is largely dependent on the NOX2 phagocyte oxidase.

### Perturbation of ubiquitin homeostasis selectively potentiates pro-inflammatory cytokine production in response to LPS stimulation

**Figure 7:**
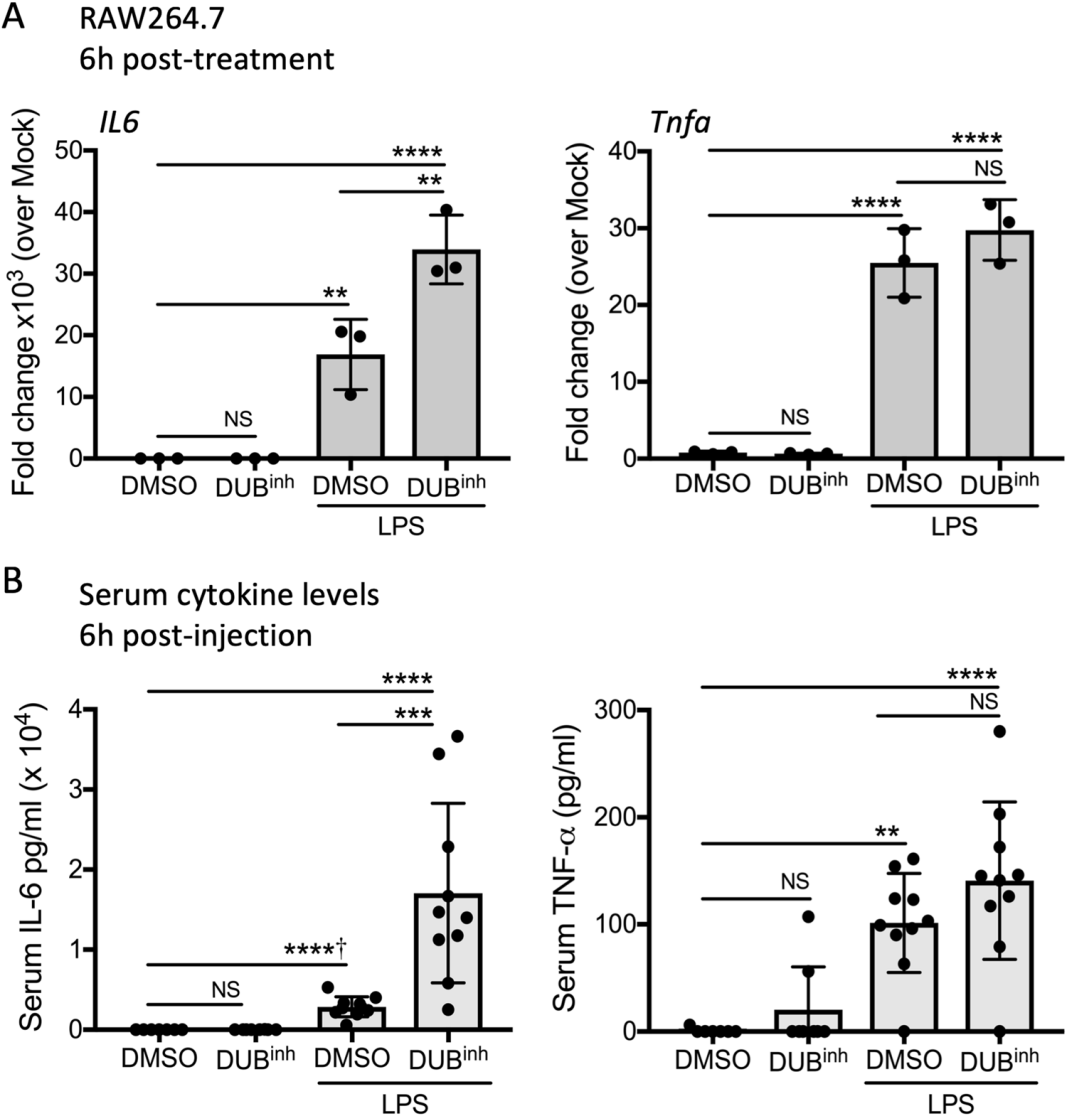
Perturbation of ubiquitin homeostasis in macrophages promotes cytokine secretion. (**A**) RAW264.7 cells treated for 0.5 h with 3.5 µM DUB^inh^, followed by 6 h of incubation with 100 ng/ml of LPS were analyzed by qRT-PCR for *Il6* and *Tnfa* transcripts. Data are from 3 independent experiments. (**B**) C56BL/6 mice were injected via intraperitoneal route with carrier control DMSO (n = 7), DUB^inh^ 20 mg/kg (n = 8), carrier control and LPS 10 mg/kg (n = 10) or DUB^inh^ and LPS (n = 10). Six hours later, sera were analyzed for IL-6 and TNF-α. Significant differences were calculated using two-tailed Student’s t test or one-way ANOVA with Tukey’s multiple comparison test (NS, not significant, **p < 0.01, ***p < 0.001, ****p < 0.0001, ^†^Student’s t test used).

Generation of ROS is one effector mechanism used by immune cells to fight invading pathogens. Infection also stimulates multiple signaling pathways leading to the release of pro-inflammatory mediators, including cytokines. To gain a broader understanding of the effect of ubiquitin perturbation in the context of immune stimulation, we looked at cytokine production in response to bacterial lipopolysaccharide (LPS), in the presence of the DUB inhibitor. As expected, RAW264.7 cells treated for 6 h with LPS exhibited a significant increase in transcription of pro-inflammatory cytokine genes, including genes encoding IL-6 and tumor necrosis factor (TNF-α) (Fig. 7A). Notably, co-treatment of macrophages with DUB^inh^ and LPS resulted in a 2-fold increase in *il6* mRNA transcripts over LPS alone, whereas *tnfa* transcript levels were not affected. Treatment with DUB^inh^ alone was not sufficient to induce pro-inflammatory cytokine genes (Fig. 7A). We next investigated the physiological relevance of these observations by looking at *in vivo* cytokine production in response to LPS. Upon intraperitoneal administration of a sub-lethal dose of LPS, mice produced high levels of serum TNF-α and IL-6, whereas mice injected with DUB^inh^ alone showed no significant increase in cytokine production (Fig. 7B). Co-injection of LPS and DUB^inh^ potentiated IL-6 production in mice, suggesting that perturbation of ubiquitin homeostasis can act in synergy with immune stimulation to selectively amplify the inflammatory response.

## Discussion

Recent studies indicate that cells recognize sudden perturbations of normal cellular functions as a signal to activate innate immune defenses^3,5^. Here, we propose a new homeostasis-associated molecular pattern, perturbation of ubiquitin homeostasis, that can be sensed by macrophages as a trigger for antimicrobial activity. As microbial infections can change the ubiquitin landscape of cells, and multiple bacterial and viral pathogen genomes encode proteins that hijack the host ubiquitination machinery, it is conceivable that cells possess mechanisms to detect these changes. Therefore, we used a chemical biology approach to cause global alterations in the ubiquitin proteome in order to study specific macrophage responses. Treatment with either a DUB inhibitor or the p97 modulator EerI resulted in increased polyubiquitinated proteins, including K48-, K63- and M1- specific linkages, validating our approach. Notably, we observed that perturbation of ubiquitin homeostasis promotes a robust but transient NOX2-dependent burst of ROS and potentiates the production of the inflammatory cytokine IL-6 in response to LPS, suggesting that perturbation of ubiquitin homeostasis might be a key signal to amplify innate immune defense mechanisms.

Proteomic studies have highlighted major differences in the repertoire of changes to the cellular ubiquitin proteome that depend on the trigger used to perturb ubiquitin dynamics. Proteasome and p97 inhibition, both resulting in increased global polyubiquitinated protein accumulation, elicit unique and distinct alterations to the ubiquitin-modified proteome^12^. Indeed, we showed here that proteasome inhibitors failed to induce ROS generation in macrophages despite their ability to alter ubiquitin homeostasis in cells. Moreover, a specific inhibitor of a proteasome-associated DUB, USP14, failed to induce ROS generation, suggesting that the DUB or DUBs targeted by the DUB^inh^ are required for regulatory functions beyond targeting proteins to the proteasome. In light of our results, it is tempting to hypothesize that altering p97 or DUB activity could trigger anti-microbial activity by altering ubiquitination events that control protein function in a regulatory, rather than a degradative way.

In phagocytic cells, the best-known function of NOX2-dependent ROS is antimicrobial activity^55^. Accordingly, we showed that ROS generated upon DUB inhibition results in an increased capacity of macrophages to control viral and bacterial intracellular infections. However, NADPH-generated ROS can also act as second messengers important for signaling and trigger other macrophage effector functions, including autophagy and inflammasome activation^56,57^. *In vivo*, NADPH-generated ROS are also key signaling molecules for immune cell recruitment at wound or infection sites and are involved in modulation of adaptive immune responses^57,58^. Thus, generation of ROS by macrophages following global alteration of the ubiquitin landscape might be a strategic way to trigger multiple effector mechanisms that would enhance protection against a broad spectrum of pathogens.

In order to establish conditions favorable for survival and proliferation in a host, pathogens employ multiple strategies, including modification of host signaling pathways essential for cell survival^59,60^. It is therefore advantageous for the cell to guard fundamental processes. This concept of a “guard model” has been well studied in plant immunity, where PTM of guard proteins is a common sensing mechanism, highlighting the importance of PTMs in this paradigm. Recently, a molecular basis for this concept was established in mammalian cells using macrophages. The pyrin inflammasome is activated upon loss of pyrin phosphorylation resulting from RhoA small GTPase inactivation by pathogens^61^. Therefore, similar to the guard proteins in plant immunity, the mammalian pyrin inflammasome acts as a global sensor modulated by PTMs. However, the possible guard sensors identified so far in mammalian cells are limited. A classic HAMP immune sensor, as predicted by the model, would respond to perturbation of cellular homeostasis, and we showed here that disruption of ubiquitin dynamics induced macrophage antimicrobial effector function. It is tempting to speculate that macrophages possess specific HAMP sensors responsive to an alteration of the ratio of free ubiquitin to ubiquitin conjugates and/or specific ubiquitin linkages. Another possibility would be a sensor that interacts or loses its interaction with p97 when there is an overabundance of ubiquitinated substrates. However, further study will be required to determine a guard sensor for ubiquitin homeostasis perturbation and the exact nature of the signal triggering its activation.

By using compounds targeting two distinct cellular processes that change the global ubiquitin landscape, we show here that alteration of ubiquitin status can be a trigger for macrophages to activate innate immune functions. This model of HAMP-dependent macrophage signaling may enhance protection against microbial infection but may also be relevant to the development of some autoimmune diseases. Indeed, substantial alterations in cellular homeostasis have been linked with many conditions associated with increased inflammation, including obesity, hypertension, Type 2 diabetes, and atherosclerosis^62^. Taken together, our results lead us to propose global ubiquitin perturbation as a potent signal that triggers an acute defense response in macrophages with the potential to control a broad spectrum of pathogenic infections.

## Materials and Methods

### Reagents

DUB^inh^-related compounds were dissolved in dimethyl sulfoxide (DMSO), aliquoted and stored at −80 °C. The two DUB inhibitors, DUB^inh^ (previously called G9 or compound 9) and DUB^inh^ C6, used in this study have been described elsewhere^29,30^. The DUB inhibitor G9 is used extensively in this study and therefore is called DUB^inh^ throughout the manuscript. The DUB inhibitor C6 was used to confirm results and is labelled as DUB^inh^ C6 in the text. Full experimental details around the synthesis of DUB^inh^, DUB^inh^-biotin, and ΔCN-biotin as well as additional reagent descriptions are described in Supplementary Information.

### Bacterial strains and viruses

*L. monocytogenes* 10403 S was grown in brain hearth infusion (BHI) broth statically at 30 °C overnight. The plaque-purified MNV-1 strain (GV/MNV1/2002/USA) MNV1.CW3 (referred as MNV-1 in the text) was used at passage 6 for all experiments^63^.

### Mice

Wild-type C57BL/6 Mice were housed in specific pathogen-free facilities, maintained by the Unit for Lab Animal Medicine of the University of Michigan. This study was carried out in accordance with the recommendations in the guide for the care and use of laboratory animals of the National Institutes of Health and the protocol was approved by the committee on the care and use of animals of the University of Michigan. NOX2-deficient (*cybb*^−/y^), *Nox1*- and *Nox4*- deficient mice and age-matched wild-type C57BL/6 mice were purchased from Jackson Laboratories. For cytokine analysis, C56BL/6 female mice (8–9 weeks) were injected intraperitoneally with control carriers DMSO and PBS (n = 7), DUB^inh^ 20 mg/kg and PBS (n = 8), DMSO and LPS (ultrapure, cat# tlrl-smlps, InvivoGen) 10 mg/kg (n = 10) or DUB^inh^ and LPS (n = 10). Blood was collected 6 h post-injection by heart puncture. Serum was extracted from blood by centrifugation at 8,000 × g for 3 minutes and used to measure level of IL-6 and TNF-α by ELISA (Cancer Center Immunology Core, University of Michigan). Mice experiments were randomized but not blinded.

### Cell culture

Bone-marrow derived macrophages (BMDM) were prepared by flushing mouse femurs in Dulbecco’s modified Eagle’s medium (DMEM) medium. Differentiation was done by incubating cells in BMDM media containing 50% DMEM, 30% L929-conditioned medium, 20% heat-inactivated fetal bovine serum (FBS), 5% L-Glutamine, 0.05% β-mercaptoethanol and 100 units/ml of penicillin-streptomycin (Pen/strep) for six days. To generate immortalized BMDM (iBMDM), freshly harvested bone-marrow cells were transduced with the J2 retrovirus and differentiated in macrophages as above^64^. iBMDM were maintained in DMEM medium containing 10% FBS, 5% L-Glutamine, 1% non-essential amino acids (NEAA) and 1% HEPES. RAW264.7 cells were grown in DMEM supplemented with 10% FBS, 5% L-Glutamine, 1% NEAA, 1% HEPES and Pen/Strep, whereas THP-1 and U937 cells were grown in RPMI 1640 medium supplemented with 10% FBS, 5% L-Glutamine, 1% NEAA, 1% HEPES, 0.05% β-mercaptoethanol and Pen/Strep.

### Isolation of peritoneal and splenic macrophages

Peritoneal macrophages were harvested after washing the peritoneal space twice with 3-ml of cold PBS. Splenocytes were obtained by passing the spleen through a 70 µM cell strainer. Red blood cells were removed by incubation of splenocytes at room temperature for 2 minutes in lysis buffer (155 mM ammonium chloride, 12 mM sodium chloride and 0.1 mM EDTA pH 8). Splenocytes were stained with 1 µg of phycoerythrin (PE) rat anti-mouse F4/80 antibody (BD biosciences #565410) in order to label the macrophage population.

### Protein extraction, pull down and immunoblotting

For pull-down experiments using DUB^inh^-biotin probe, RAW264.7 cells were seeded at a density of 7 × 10^6^ cells per 100 mm dish and allowed to adhere overnight. Cells were washed twice with Dulbecco’s phosphate-buffered saline (PBS) and lysed in ice-cold cell lysis buffer (1% NP-40, 150 mM NaCl, 50 mM Tris-Hcl pH 8, 1 mM EDTA pH 8, 0.1% SDS, 0.5% sodium deoxycholate, 1X Roche protease inhibitor). After 15 minutes incubation on ice, cells were briefly sonicated and split into 4 tubes. The input tube was diluted in 4X concentrated sample buffer (BioRad) containing β-mercaptoethanol and denatured for 10 minutes at 95 °C. The remaining tubes were either left untreated (mock sample) or incubated at 37 °C for 30 minutes with 20 µM DUB^inh^-biotin or ΔCN-biotin. Pre-washed streptavidin agarose resin (Pierce) was added directly to the lysate and tubes were incubated with agitation for 2 h at 4 °C. Agarose beads were washed 6 times with lysis buffer and resuspended in 75 µl of 1 X sample buffer. To obtain whole-cell lysates, RAW264.7 cells or iBMDM cells were seeded in 6-well plates at an initial density of 6 × 10^6^ cells per plate. iBMDM were activated overnight with 100 ng/ml of LPS and interferon-γ. After treatment described in the specific figure legends, cells were lysed in a cell lysis buffer (10 mM Tris-HCl pH 8, 150 mM NaCl, 1% NP-40, 10 mM EDTA pH 8, 1 mM DTT and 1X Roche protease inhibitors), incubated on ice for 15 minutes, quickly sonicated and diluted in 4X sample buffer. Samples were separated by SDS-PAGE and transferred to polyvinyldene fluoride membrane (PVDF, Millipore). Immunoblotting was performed according to the antibody manufacturers’ instructions.

### *L. monocytogenes* intracellular growth

RAW264.7 cells were seeded at a density of 6 × 10^6^ cells per 24-well tissue-culture plate and allowed to adhere overnight. iBMDM were seeded at a density of 4 × 10^6^ cells per 24-well plate, allowed to adhere for 4 hours and then activated overnight with 100 ng/ml of LPS and interferon-γ. Where indicated, cells were pre-treated with 10 mM NAC, 500 µM L-glutathione reduced (GSH), 10 µM GSK-PERK inhibitor or 50 µM 4µ8c Ire1 inhibitor for 30 minutes followed by a 30-minute treatment with 3.5 µM of DUB^inh^ or equivalent volume of DMSO (vehicle control). DUB^inh^ were removed from cells by changing the medium. Macrophages were then infected with *L. monocytogenes* at a multiplicity of infection (MOI) of 1 for 30 minutes. The inoculum was removed by washing cells with PBS three times, and cells were incubated in medium containing 10 µg/ml of gentamicin. Cells were collected at 6 h p.i. in 1 ml of 0.1% Triton X-100, serially diluted and plated on Luria-Bertani (LB) agar for enumeration. Colony forming unit (CFU) were counted using the Acolyte plate reader and software (Microbiology International). Results were normalized to DMSO-treated cells.

### Virus infection and plaque assay

For all MNV infections, RAW264.7 cells were seeded in 12-well plates at 5 × 10^5^ cells/well and grown overnight. Cells were incubated for 30 minutes at 37 °C with 1 mL medium containing no reagent, 2.5 μM C6, or equivalent volume of DMSO as vehicle control. Then cells were infected with MNV-1 (CW3 at pass 6) at MOI 5 and rocked on ice for 1 hour for viral adherence. Cells were then washed 3 times with ice cold PBS and warm medium was returned to the cells with no reagents, 2.5 μM C6, or equivalent volume of DMSO. For NAC- and L-glutathione-treated samples, NAC was added to a final concentration of 10 mM and Glutathione at a final concentration of 500 μM in addition to C6 or DMSO after viral adherence on ice. Cells were then incubated at 37 °C for 8 hours. Plates were frozen at −80 °C overnight, then freeze-thawed twice. Plaque forming units measured by plaque assay as described before^65^. For EerI experiments, cells were infected exactly as with C6 above, but at a concentration of 5.0 μM both before and after MNV infection.

### RNAi knockdown

SiGENOME Smart Pool small interfering RNA (siRNA) for mouse p97/vcp and the non-targeting siRNA were purchased from Dharmacon, and the manufacturer’s protocol was followed for the delivery of siRNA into the cells. Briefly, RAW264.7 cells were seeded in 24-well plates at 1.5 × 10^6^ cells/well and grown overnight. Transient knockdown was done by transfecting the cells using the DharmaFECT reagent 4 (Dharmacon) and a final concentration of 50 nM siRNA per well. The medium was replaced 6 h post-transfection. The cell viability was assessed at different time points after transfection by diluting 1 in 10 the WST-1 reagent (Roche) in the media. Cells were incubated for 1.5 h before reading the optical density of the supernatant at 440 and 600 nm. Results were normalized to NT siRNA-transfected cells.

### Quantitative real-time PCR

RNA from RAW264.7 cells treated with 3.5 µM DUB^inh^ (or equivalent volume of DMSO) followed by 6 h incubation with LPS 100 ng/ml were isolated using RNeasy kit from Qiagen. cDNA were generated using 1 µg of RNA and quantitative real time PCR analysis was preformed using CFX96 real time system (Biorad). The sequences of the primers used were: F: 5′-CCTCTGGTCTTCTGGAGTACC-3′ and R: 5′-ACTCCTTCTGTGACTCCAGC-3′ for IL-6; F: 5′-ATGAGCACAGAAAGCATGA-3′ and R: 5′-AGTAGACAGAAGAGCGTGGT-3′ for TNFα; F: 5′-ATGGTGGGAATGGGTCAGAAGGAC-3′ and R: 5′-CTCTTTGATGTCACGGACGATTTC-3′ for actin (internal control). Results were normalized to the level of actin mRNA and we applied the comparative ΔΔCt (ΔCt _target_ − ΔCt _control_) method for analysis.

### ROS measurements

Macrophages were pre-treated with 10 mM NAC, 500 µM GSH or 300 µM TUDCA for 30 minutes, followed by a 0.5 h treatment with 3.5 µM of DUB^inh^ or equivalent volume of DMSO. Alternatively, cells were treated with EerI (10 µM), MG132 (5 µM), epoxomicin (0.5 µM) or IU1 (100 µM) for 1 h. Staining was done for 0.5 h with 5 µM CM-H_2_DCFDA (general ROS indicator) in HBSS buffer, followed by 0.5 h incubation with fresh media. For time course experiments, cells were drug-treated as above, washed and then incubated in fresh medium for the indicated time before staining. For multiple challenges with DUB^inh^, cells were treated with 3.5 µM DUB^inh^ for 0.5 h, washed, incubated in fresh media for 4 h and stimulated again with 3.5 µM DUB^inh^ before staining with 5 µM CM-H_2_DCFDA. Peritoneal macrophages and splenocytes were stained with 1.25 µM or 2 µM of CM-H_2_DCFDA, respectively. Live cells were gated based on the forward and side scatter profile. Fluorescence was read on Fortessa or Canto flow cytometers (BD Biosciences) and data were further analyzed using FlowJo software. For all experiments using EerI, the intrinsic fluorescence of EerI was subtracted for calculation of the MFI.

### Statistical analysis

Results represent the mean and the corresponding standard deviation of the mean for at least three independent experiments (unless specified) as indicated in figure legends. Statistical analysis was performed using GraphPrism 7 software and unpaired, two-tailed student’s t test or one-way analysis of variance (ANOVA) with Dunnett or Tukey’s post-tests were used, as indicated in each legend.

## Supplementary information

Supplementary Information

## Data Availability

All data presented in this article and the corresponding Supplementary Information File are available upon request from the corresponding author.
