# Supplementary material for: Perturbation of ubiquitin homeostasis promotes macrophage oxidative defenses
Source: Sci Rep. 2019 Jul 15;9:10245. doi: 10.1038/s41598-019-46526-9 (PMC6629656; doi:10.1038/s41598-019-46526-9)
Supplement: Supplementary file 1 — Supplementary Information [file 41598_2019_46526_MOESM1_ESM.pdf]

Supplementary Information for

Perturbation of ubiquitin homeostasis promotes macrophage oxidative defenses

Marie-Eve Charbonneau[1], Karla D. Passalacqua[1], Susan E. Hagen[2], Hollis D. Showalter[2], Christiane E. Wobus[1], Mary X.D. O'Riordan[1*]

[1]Department of Microbiology and Immunology, University of Michigan Medical School, Ann Arbor, Michigan, United States of America, 48109

[2]Department of Medicinal Chemistry, College of Pharmacy, University of Michigan, Ann Arbor, Michigan, United States of America, 48109

*Corresponding author: Mary O'Riordan, Dept. of Microbiology & Immunology, University of Michigan Medical School, 5641 Medical Sciences II, 1150 W. Medical Ctr Dr, Ann Arbor, MI, 48109, 734-615-4289, oriordan@umich.edu

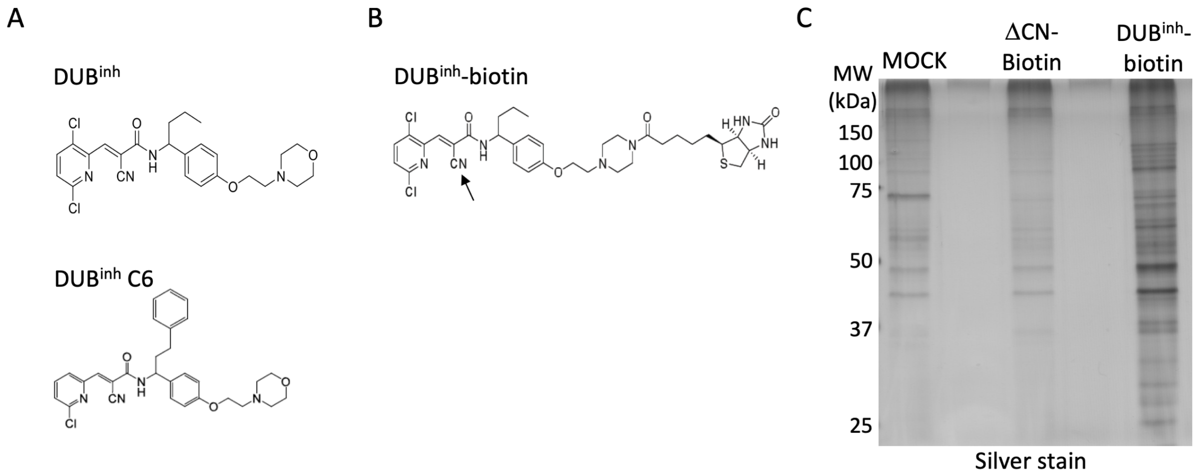

Fig. S1: DUB^{inh} used in this study. A) Structure of the DUB inhibitors DUB^{inh} and DUB^{inh} C6. Structure of the DUB^{inh}-biotin compound. The arrow highlights the key cyano group. B) RAW264.7 whole cell lysates were incubated with DUB^{inh}-Biotin or ΔCN-biotin before immunoprecipitation using streptavidin-coated beads. The retained proteins were revealed by silver stain. Data are representative of three independent experiments. Full-length gel is shown in Figure S7A.

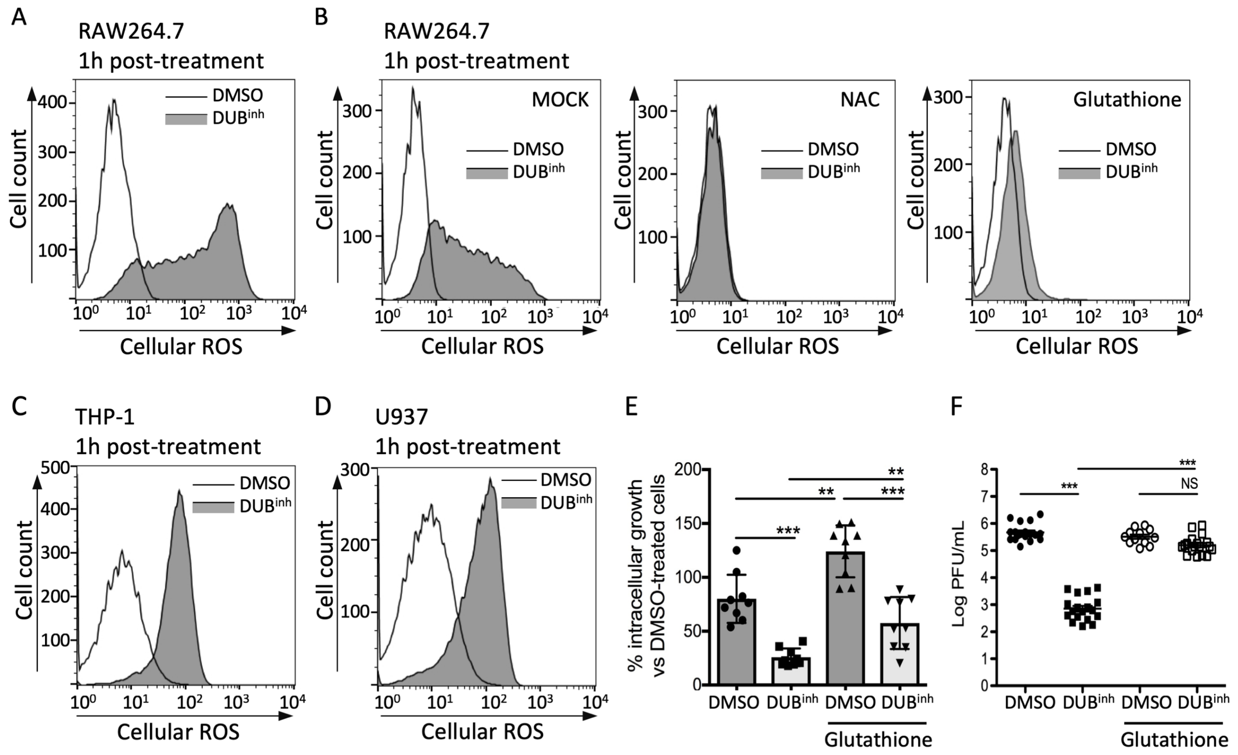

Fig. S2: Characterization of DUB^{inh} induces ROS generation in mouse and human cell lines. A) FACS analysis of RAW264.7 cells treated with 2.5 μM DUB^{inh} C6 for 0.5h before staining with 5 μM of the general ROS dye CM-H_2DCFDA. B) RAW264.7 cells were incubated with 5 mM NAC, 100 μM L-reduced glutathione or medium only for 0.5h. DUB^{inh} (or equivalent volume of DMSO) was added at a final concentration of 3.5 μM for 0.5h before staining for ROS detection. C) and D) FACS analysis of THP1 and U937 human monocytes treated with DUB^{inh} for 0.5h before staining with 5 μM of CM-H_2DCFDA. All FACS plots are representative of at least three independent experiments. E) RAW264.7 cells were incubated with 100 μM L-reduced glutathione or medium only for 0.5h, followed by incubation with DUB^{inh} (or equivalent volume of DMSO) at a final concentration of 3.5 μM for 0.5h. Cells were then infected with *L. monocytogenes* (MOI 1) for 0.5h. washed and new medium containing 10 μg/ml of gentamicin was added. Intracellular bacteria were enumerated at 6 h p.i. The data from three independent experiments represent percent of intracellular *L. monocytogenes* versus DMSO-only treated cells. F) RAW264.7 cells were pre-treated with 100 μM L-reduced glutathione or medium in combination with 2.5 μM DUB^{inh} C6 or equivalent volume of DMSO for 0.5h before infection with MNV-1 (MOI 5) for 1h on ice. Cells were collected at 8 h p.i. and viral titers were determined by plaque assay. Significant differences were calculated using one-way ANOVA and Tukey's multiple comparison test on the unmodified data (NS, not significant, ** $p < 0.01$, *** $p < 0.001$).

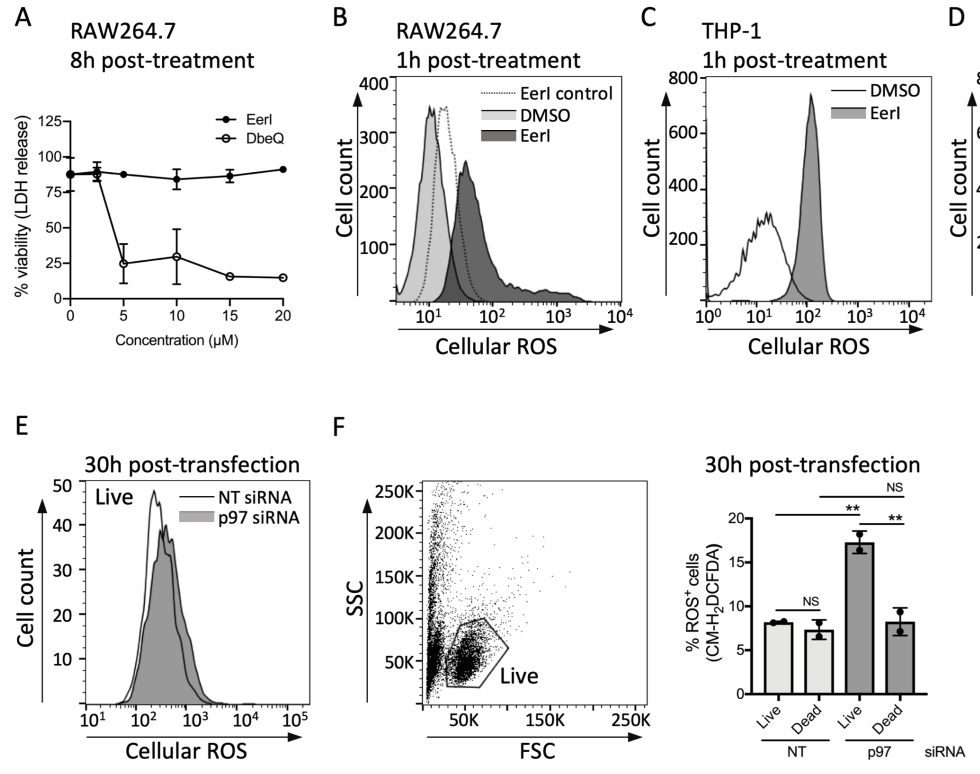

Fig. S3: Alteration of p97 function induces ROS generation. A) Cellular viability measured by the LDH release in the media at 7.5h post-treatment with increasing concentrations of EerI and DBeQ. B) FACS analysis of RAW264.7 cells treated for 1h with 10 µM EerI and stained with 5 µM CM-H_2DCFDA. The EerI control histogram represents cells treated with EerI for 1h but not stained with the ROS dye. FACS analysis of THP1 (C) and U937 (D) human monocytes treated with 10 µM EerI for 1h before staining with 5 µM CM-H_2DCFDA. FACS plots are representative of at least three independent experiments. E) At 30h post-transfection with siRNA against p97 or a non-targeted control, cells were stained with 5 µM CM-H_2DCFDA for 0.5h. FACS plots are representative of 2 independent experiments. F) FSC and SSC plot (left panel) of RAW264.7 cells at 30h post-transfection with a non-targeted control siRNA showing the Live population used to measure ROS production. Quantification of percentage of cells stained for ROS (% ROS^+ cells) at 30h post-transfection (right panel) comparing the ROS production in live and dead cells. Significant differences were calculated using one-way ANOVA and Tukey's multiple comparison test on the unmodified data (NS, not significant, ** $p < 0.01$).

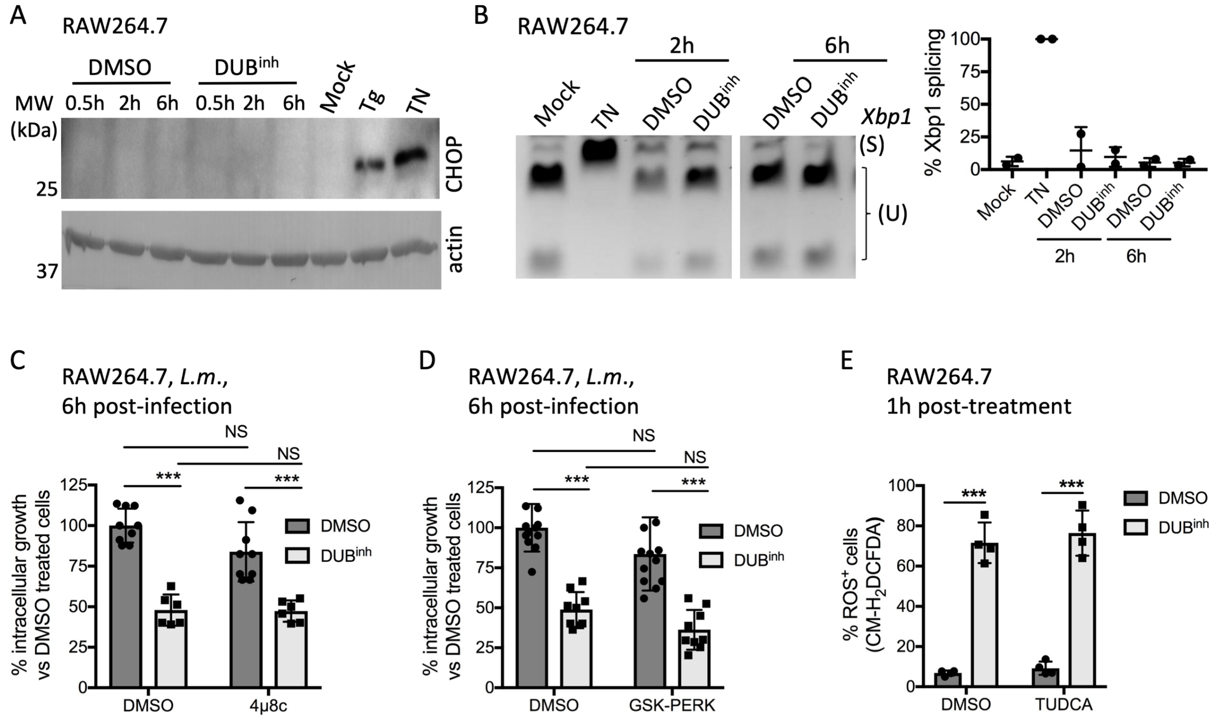

Fig. S4: DUB^{inh} induces ROS generation independently of UPR signaling. A) RAW264.7 cells were treated for 0.5h with 3.5 µM DUB^{inh}. Following treatment, cells were washed and subsequently incubated for the indicated period of time in fresh medium. As control, cells were treated with 10 µM thapsigargin or 10 µg/ml of tunicamycin for 4h. Whole cells lysates were used for immunoblotting against CHOP or actin as a loading control. B) RAW264.7 were treated as above. PCR was performed to amplify the *Xbp1* mRNA and products were digested with the Pst1 endonuclease. The unspliced (U) form of *Xbp1* contains a Pst1 cleavage site, which will generate two smaller fragments compared to the *Xbp1* spliced (S) form. The percentage of *Xbp1* splicing was calculated by band densitometry as follow: *Xbp1*(S) / [*Xbp1*(U)+*Xbp1*(S)]. Results for A) and B) are from two independent experiments. RAW264.7 cells were incubated for 0.5h with 50 µM 4µ8C C) or 50 µM GSK-PERK D). DUB^{inh} (or equivalent volume of DMSO) was added on top at a final concentration of 3.5 µM for 0.5h. After incubation, the medium was removed, and cells were infected with *L. monocytogenes* (MOI 1) for 0.5h. Following infection, cells were washed and new medium containing 10 µg/ml of gentamicin was added. Intracellular bacteria were enumerated at 6h p.i. The data represent percent of intracellular *L. monocytogenes* growth compared to DMSO-only treated cells. Results were obtained from three independent experiments performed in triplicate E) RAW264.7 cells were incubated with 300 µM TUDCA or medium only for 1h. DUB^{inh} (or equivalent volume of DMSO) was added on top at a final concentration of 3.5 µM for 0.5h before staining for ROS detection. The results from four experiments represent the percentage of cells stained for ROS (% ROS^{+} cells). Significant differences were calculated using one-way ANOVA and Tukey's multiple comparison test on the unmodified data (NS, not significant, *** $p < 0.001$). Full-length immunoblots and gel are shown in Figure S8A and B respectively.

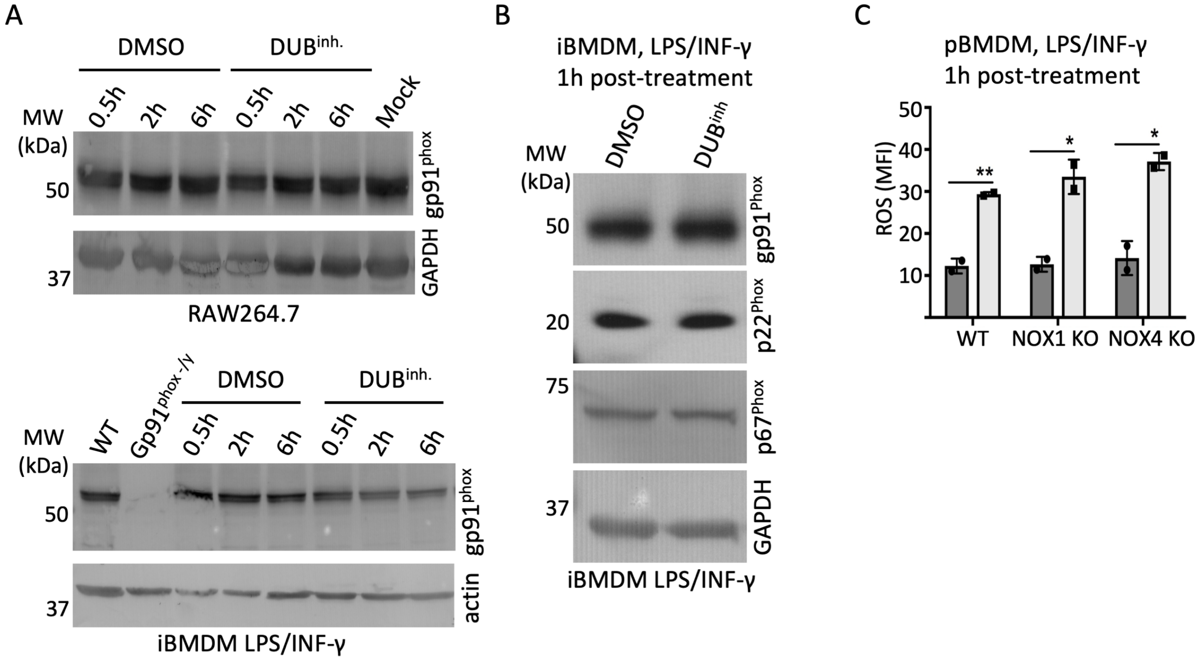

Fig. S5: DUB inhibition induces ROS generation through NOX2 complex without affecting protein expression levels of gp91, p22 or p67. A) RAW264.7 cells or WT and gp91$^{phox-/y}$ iBMDM incubated overnight with 100 ng/ml of LPS and INF-γ were treated for 0.5h with 3.5 μM DUB^{inh}. Following treatment, cells were washed and subsequently incubated for the indicated period of time in fresh medium. Whole cells lysates were used for immunoblotting against gp91^{phox} and GAPDH or β-actin as loading controls. Results are from 2 independent experiments. Full-length blots are presented in supplementary figure 8C. B) WT iBMDM incubated overnight with 100 ng/ml of LPS and INF-γ were treated for 0.5h with 3.5 μM DUB^{inh}. Following treatment, cells were washed and subsequently incubated for 0.5h. Whole cells lysates were used for immunoblotting against gp91^{phox}, p22^{phox}, p67^{phox} and GAPDH as loading controls. C) pBMDM isolated from WT, *Nox*$^{-/-}$ (NOX1 KO) or *Nox4*$^{-/-}$ (NOX4 KO) mice and incubated overnight with 100 ng/ml of LPS and INF-γ were treated for 0.5h with 3.5 μM DUB^{inh} or equivalent volume of DMSO before staining for 0.5h with the ROS dye. Quantification of mean fluorescence intensity (MFI) from 2 independent experiments was calculated using FlowJo software. Full-length immunoblots are shown in Figure S8C and D.

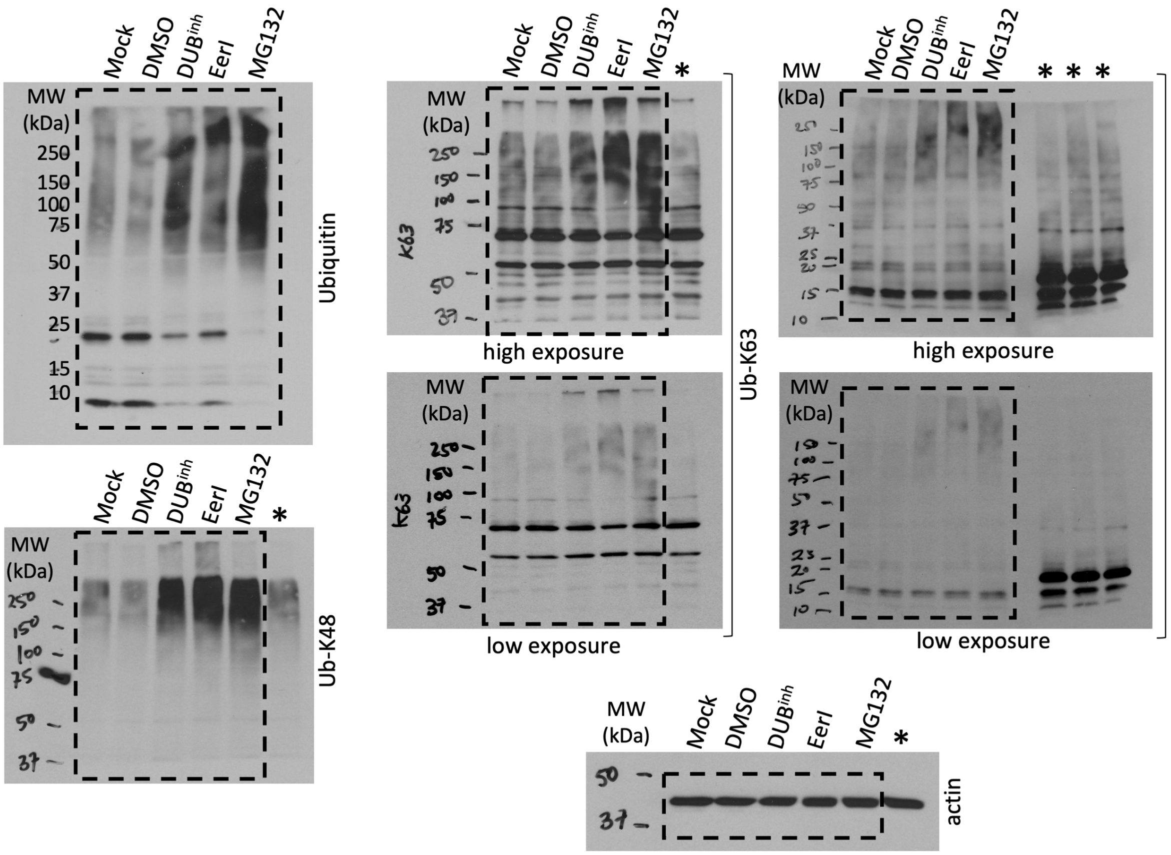

Fig. S6: Full immunoblot exposures for results shown in Figure 1A. Lines highlighted by a star represent sample unrelated to this study.

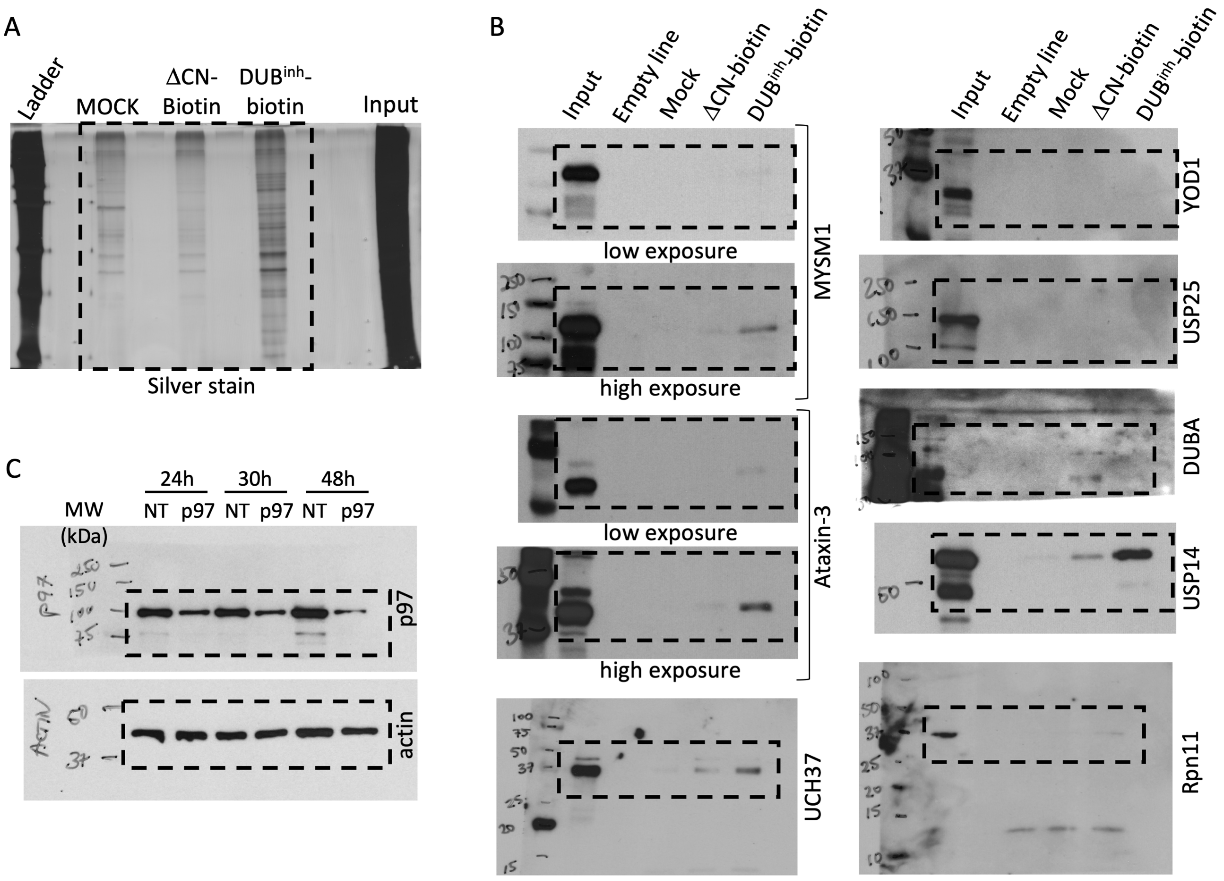

Fig. S7: A) Complete gel for results shown in Figure S1C. B) Full length immunoblots for results shown in Figure 1B. C) Full length immunoblots for results shown in Figure 4F.

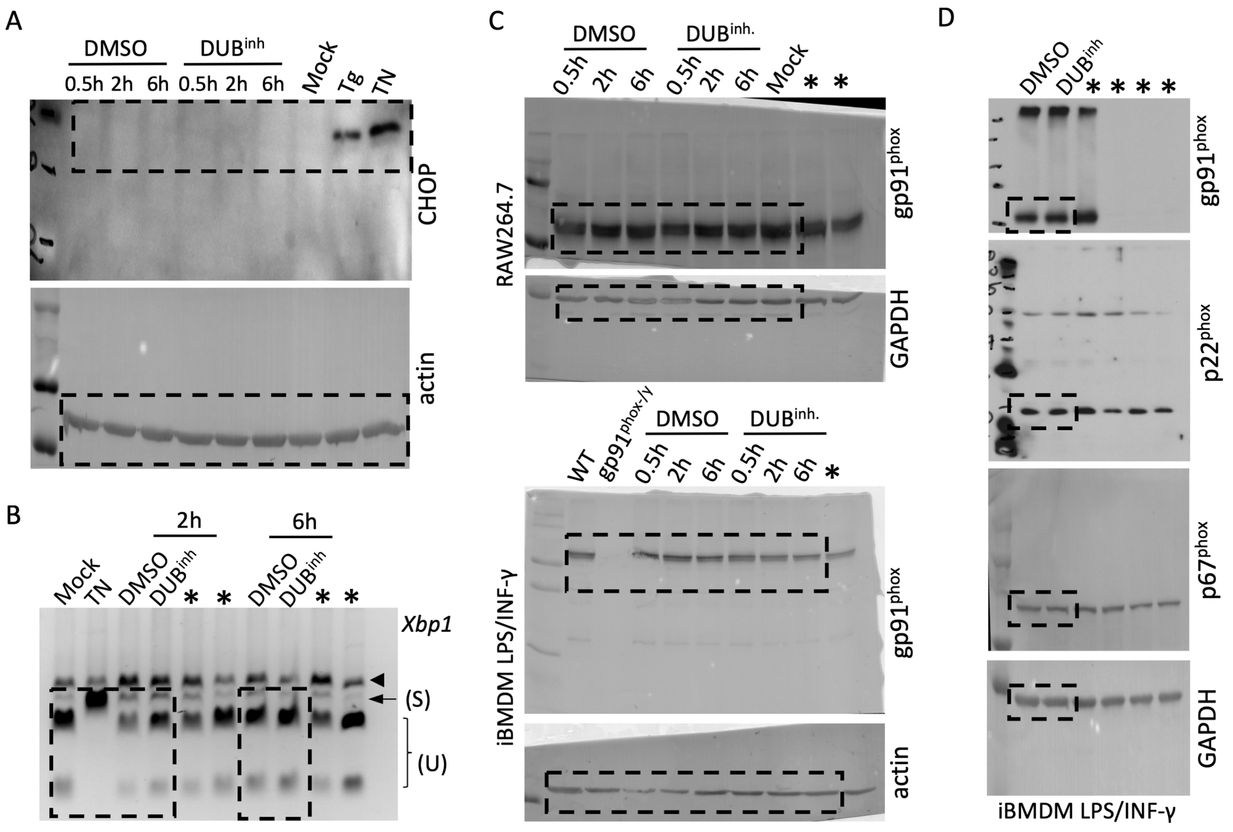

Fig. S8: A) Full length immunoblots for results shown in Figure S4A. B) Full length gel for results shown in Fig. S4B. The top band (arrowhead) represents hybrid product between spliced and unspliced PCR fragments. C) Full length immunoblots for results shown in Figure S5A. D) Full length immunoblots for results shown in Figure S5B. Lines highlighted by a star represent sample unrelated to this study.

Supplementary material and methods

Antibodies and reagents. DUB^{inh}, DUB^{inh}-biotin and ΔCN-biotin were synthesized by the University of Michigan Vahlteich Medicinal Chemistry Core. The library numbers for each compound from the University of Michigan (CCG) database are CCG-257343 for DUB^{inh}, CCG-257202 for DUB^{inh}-biotin and CCG-257203 for ΔCN-biotin. The details around the synthesis of these compounds are given below. Eeyarestatin I, IU1, DBeQ, GSK2606414 (GSK-PERK), 4μ8C, Tauroursodeoxycholic acid (TUDCA) and thapsigargin were purchased from Millipore. MG132 was purchase from Selleckchem and epoxomicin from Cayman. The general ROS indicator CM-H_2DCFDA was purchased from ThermoFisher, whereas reduced L-glutathione, WST-1 cell viability reagent and N-acetylcysteine (NAC) were purchased from Sigma. Bacterial lipopolysaccharide (LPS) was purchased from Abcam and recombinant murine interferon-γ from Peprotech. The primary antibodies used for immunoblotting were anti-ubiquitin, clone P4D1 (sc-8017), anti-USP14 (sc-100630), anti-USP25 (sc-398414), anti-GAPDH (sc-32233), $p22^{phox}$ (sc-20781) and anti-$gp91^{phox}$ (sc-130543) from Santa Cruz, anti-YOD1 (ARP67915) from Aviva Systems Biology, anti-UCH37 (A304-099A) form Bethyl Laboratories, anti-Otud5 (DUBA, 21002-1-AP) from ProteinTech, anti-MYSM1 (ab193081), anti-Ataxin3 (ab175262) and anti-$p67^{phox}$ (ab109366) from Abcam, anti-ubiquitin lys48-specific (05-1307) and anti-linear ubiquitin clone LUB9 (MABS451) from EDM Millipore, anti-ubiquitin k63-specific (5621) and anti-CHOP (2895) from Cell Signaling, anti-actin clone ACTN05 (MS-1295), anti-POH1 (Rpn11; 38-0200) and anti-p97/VCP (MA3-004) from ThermoFisher. All HRP-conjugated secondary antibodies were from Jackson ImmunoResearch. The silver stain was done using the Pierce silver stain kit (ThermoFisher).

Cell viability. RAW264.7 cells were seeded at a density of 3.5×10^4 cells per well in a 96-well plate and allowed to adhere overnight. Cells were treated with increasing concentrations of EerI or DBeQ for 7.5h. LDH release assay (Cytotox 96; Promega) was performed according to the manufacturer's instructions.

***Xbp1* splicing assay.** RNA was extracted using the RNeasy Mini Kit (QIAGEN) from samples treated with 10 μg/ml of tunicamycin for 4h or with 3.5μM DUB^{inh} or equivalent volume of DMSO for the indicated time. cDNA synthesis was performed using 1 μg of RNA and *Xbp1* transcripts were amplified using following primers: forward 5′-GAACCAGGAGTTAAGAACACG-3′ and reverse 5′-AGGCAACAGTGTCAGAGTCC-3′. Amplification was done using using 30 cycles at 94°C for 1 min, 60°C for 1 min and 72°C for 1 min. PCR products were further digested with the restriction enzyme PstI and loaded onto a 3% agarose gel to differentiate between the spliced and unspliced forms of *Xbp1*.

Methods for compound synthesis

All starting monomers were obtained from commercial suppliers and used without further purification. Routine ^{1}H NMR spectra were recorded at 400 MHz on a Varian 400, Varian 500, or Bruker 400 Avance

instrument with chloroform-*d* or DMSO-d_6 as solvent. Chemical shift values are recorded in δ units (ppm). Mass spectra were recorded on a Micromass TofSpec-2E Matrix-Assisted, Laser-Desorption, Time-of-Flight Mass Spectrometer in a positive ESI mode (TOFES$^+$) unless otherwise noted. High resolution mass spectrometry (HRMS) analysis was performed on an Agilent Q-TOF system. Analytical HPLC was performed on an Agilent 1100 series instrument with an Agilent Zorbax Eclipse Plus C18 (4.6 mm × 75 mm, 3.5 μm particle size) column with the gradient 10% acetonitrile/water (1 min), 10−90% acetonitrile/water (6 min), and 90% acetonitrile/water (2 min) flow = 1 mL/min. Thin-layer chromatography (TLC) was performed on silica gel GHLF plates (250 μm) purchased from Analtech. Column chromatography was carried out in the flash mode utilizing silica gel (220−240 mesh) purchased from Silicycle. Extraction solutions were dried over anhydrous sodium sulfate or magnesium sulfate prior to concentration. Combustion analyses were carried out by Robertson Microlit Laboratories, Ledgewood, NZ.

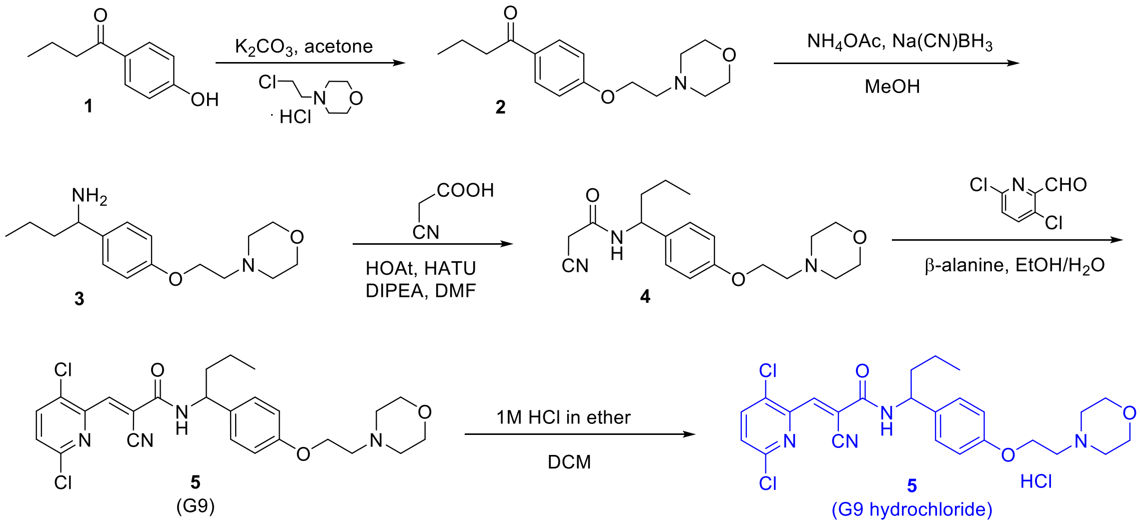

Synthetic scheme for compound G9 (DUB^{inh}) hydrochloride (**5**)

1-(4-(2-Morpholinoethoxy)phenyl)butan-1-one (2). *N*-(2-Chloroethyl)morpholine hydrochloride (34 g, 0.183 mol) was added to a stirred solution of *p*-hydroxybutyrophenone (**1**; 10 g, 0.061 mol) in acetone (58 mL) followed by the addition of potassium carbonate (33.7 g, 0.243 mol). The resulting mixture was heated at reflux for 48 h with progress of the reaction monitored by TLC (ethyl acetate:hexanes 1:1, R_f 0.3). The mixture was concentrated and diluted with ethyl acetate, and then washed with water (2x) and brine, dried, and concentrated to obtain an oil. Purification by column chromatography, eluting with ethyl acetate:hexanes (1:1), afforded **2** (12.4 g, 73%) as a clear oil. ^{1}H NMR (400 MHz, chloroform-*d*) δ 7.94 (d, *J* = 8.9, 2H), 6.94 (d, *J* = 8.9, 2H), 4.17 (t, *J* = 5.7, 2H), 3.74 (m, 4H), 2.89 (t, *J* = 7.4, 2H), 2.82 (t, *J* = 5.7, 2H), 2.58 (m, 4H), 1.76 (m, 2H), 1.00 (t, *J* = 7.4, 3H).

1-(4-(2-Morpholinoethoxy)phenyl)butan-1-amine (3). Sodium cyanoborohydride (13.37 g, 0.212 mol) was added to a stirred suspension of compound **2** (11.8 g, 0.043 mol) and ammonium acetate (98.5 g, 1.27 mol) in methanol (82 mL). The resulting mixture was heated at 40 ºC for 72 h. Progress of the

reaction was monitored by TLC (ethyl acetate, R_f 0.1). The mixture was filtered through celite and washed with methanol. The filtrate was concentrated and the residue diluted with ethyl acetate. The solution was washed with saturated aqueous sodium bicarbonate and brine, dried, and concentrated to give **3** (11.39 g, 96%), which was taken forward to the next step as a light yellow oil. ^{1}H NMR (400 MHz, chloroform-*d*) δ 7.22 (d, *J* = 8.3, 2H), 6.87 (d, *J* = 8.3, 2H), 4.11 (t, *J* = 5.6, 2H), 3.85 (t, *J* = 6.9, 1H), 3.73 (m, 4H), 2.80 (t, *J* = 5.6, 2H), 2.58 (m, 4H), 2.00 (br s, 2H), 1.63 (m, 2H), 1.26 (m, 2H), 0.89 (t, *J* = 7.3, 3H).

2-Cyano-*N*-(1-(4-(2-morpholinethoxy)phenyl)butyl)acetamide (**4**). A stirred ice-cold solution of 2-cyanoacetic acid (0.581 g, 6.82 mmol) in DMF (15 ml) was treated sequentially with HOAt (CAS 39968-33-7; 0.929 g, 6.82 mmol), HATU (CAS 148893-10-1; 2.6 g, 6.82 mmol), and diisopropylethylamine (2.62 ml, 15.01 mmol) After 5 min, compound **3** (1.9 g, 6.82 mmol) was added and the resultant mixture was stirred at room temperature for 6 h. Progress of the reaction was monitored by TLC (ethyl acetate: hexanes, 80:20, R_f 0.7). The mixture was diluted with water and extracted with ethyl acetate (2x). The organic layer was dried and concentrated to yield crude product which was purified by column chromatography, eluting with 1-4% methanol in dichloromethane, to afford **4** (1.2 g, 51%) as a viscous light yellow oil. ^{1}H NMR (400 MHz, chloroform-*d*) δ 7.22 – 7.17 (m, 2H), 6.91 – 6.86 (m, 2H), 6.26 (d, *J* = 8.0 Hz, 1H), 4.89 (q, *J* = 7.7 Hz, 1H), 4.10 (t, *J* = 5.7 Hz, 2H), 3.76 – 3.71 (m, 4H), 3.40 – 3.25 (m, 2H), 2.80 (t, *J* = 5.7 Hz, 2H),2.61 – 2.53 (m, 4H), 1.88 – 1.70 (m, 2H), 1.38 – 1.20 (m, 2H), 0.92 (t, *J* = 7.3 Hz, 3H). MS *m/z* 346.2 $(M+H)^+$. HPLC 98%.

(*E*)-2-Cyano-3-(3,6-dichloropyridin-2-yl)-*N*-(1-(4-(2-morpholinoethoxy)phenyl)butyl)acrylamide (5; G9 or DUB^{inh}). To a room temperature solution of compound **4** (1.05 g, 3.04 mmol) and 3,6-dichloropicolinaldehyde (963 mg, 5.5 mmol) in ethanol (16 mL) was added 3-aminopropanoic acid (β-alanine) (2.17 g, 24.32 mmol). To the stirred suspension was added slowly 12 mL of water to make a homogeneous solution and the mixture was stirred overnight with monitoring by TLC (5:95 methanol:dichloromethane). The reaction mixture was concentrated and the residue diluted with ethyl acetate. The solution was washed with saturated sodium bicarbonate and brine, dried, and concentrated to an oil that was purified by column chromatography eluting with 1 - 2% methanol in dichloromethane. Product fractions were combined to give **5** (920 mg, 60%) as a viscous yellow oil. ^{1}H NMR (400 MHz, chloroform-*d*) δ 8.61 (s, 1H), 7.74 (d, *J* = 8.5 Hz, 1H), 7.38 (d, *J* = 8.5 Hz, 1H), 7.24 (d, 2H), 6.89 (d, *J* = 8.7 Hz, 2H), 6.78 (d, *J* = 7.8 Hz, 1H), 5.01 (q, *J* = 7.6 Hz, 1H), 4.10 (t, *J* = 5.7 Hz, 2H), 3.78 – 3.71 (m, 4H), 2.80 (t, *J* = 5.7 Hz, 2H), 2.60 – 2.55 (m, 4H), 1.96 – 1.76 (m, 2H), 1.41 – 1.30 (m, 2H), 0.95 (t, *J* = 7.4 Hz, 3H); MS (ES$^-$) *m/z* 501.3 $(M – H)^-$ / 503.4 $(M - H)^-$ (3:2 Cl isotope pattern).

G9 (DUB^{inh}) hydrochloride salt formation was carried out as follows: **5** free base (910 mg) was dissolved in 5 mL of dichloromethane and the stirred solution was treated with 5.4 mL of 1N HCl in ether. After 5 min, the solvent was removed under reduced pressure to gave yellow guey residue. It was dried under high vacuum for 6 h to leave **5** hydrochloride (1.0 g, 100%) as a yellow powder. ^{1}H NMR (400 MHz, DMSO-d_6) δ 10.90 (br s, 1H), 9.02 (d, *J* = 8.2 Hz, 1H), 8.26 – 8.00 (m, 2H), 7.73 (d, *J* = 8.6 Hz, 1H), 7.33 (d, *J* = 8.6 Hz, 2H), 6.96 (d, *J* = 8.6 Hz, 2H), 4.84 (q, *J* = 8.1 Hz, 1H), 4.38 (t, *J* = 4.8 Hz, 2H), 3.94

(d, J = 12.8 Hz, 2H), 3.77 (t, J = 12.1 Hz, 2H), 3.63 – 3.28 (m, 8H, contains water), 3.17 (d, J = 11.3 Hz, 2H), 1.98 – 1.77 (m, 1H), 1.68 (td, J = 13.9, 13.3, 6.4 Hz, 1H), 1.28 (ddd, J = 28.2, 15.4, 8.2 Hz, 2H), 0.88 (t, J = 7.3 Hz, 3H). HPLC: 99.5%. *Anal.* Calcd. for $C_{25}H_{28}Cl_2N_4O_3 \cdot 0.9\ HCl \cdot H_2O$: C, 54.18; H, 5.62; N, 10.11, Cl, 18.55. Found: C, 54.07; H, 5.37; N, 9.82, Cl, 18.46. MW = 554.26.

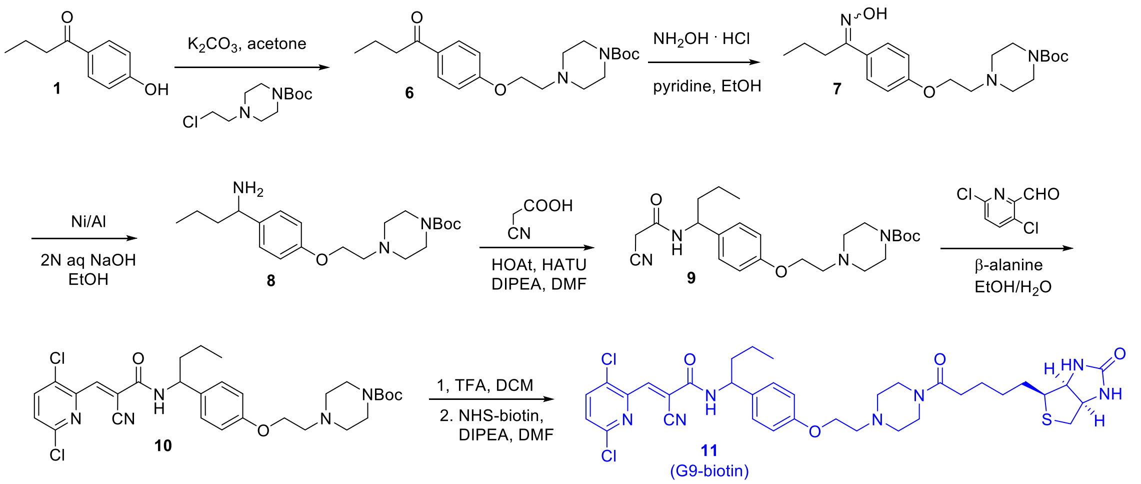

Synthetic scheme for compound G9-biotin (DUB^{inh}-biotin) (**11**)

***tert*-Butyl 4-(2-(4-butyrylphenoxy)ethyl)piperazine-1-carboxylate (6)**. A stirred suspension of compound **1** (0.585 g, 3.56 mmol), *tert*-butyl 4-(2-chloroethyl)piperazine-1-carboxylate (2.66 g, 10.7 mmol), potassium carbonate (1.97 g, 14.3 mmol) and acetone (15 mL) was heated at reflux overnight. The cooled mixture was diluted with ethyl acetate and washed sequentially with water (2x) and brine (2x), dried, and concentrated to a tan oil. Column chromatography, eluting with 1:1 hexanes:ethyl acetate, gave a yellow oil which solidified under high vacuum to give **6** (1.31 g, 98%). ^{1}H NMR (500 MHz, chloroform-*d*) δ 7.98-7.88 (m, 2H), 6.93 (d, J = 8.8 Hz, 2H), 4.16 (t, J = 5.7 Hz, 2H), 3.45 (t, J = 5.0 Hz, 4H), 2.89 (t, J = 7.3. Hz, 2H), 2.84 (t, J = 5.7 Hz, 2H), 2.53 (t, J = 5.0 Hz, 4H), 1.75 (q, J = 7.4 Hz, 2H), 1.46 (s, 9H), 0.99 (t, J = 7.4 Hz, 3H).

***tert*-Butyl 4-(2-(4-(1-(hydroxyimino)butyl)phenoxy)ethyl)piperazine-1-carboxylate (7)**. A solution of compound **6** (1.66 g, 4.41 mmol), hydroxylamine hydrochloride (0.613 g, 8.82 mmol), pyridine (1.07 mL, 13.2 mmol) and ethanol (50 mL) was stirred at 80 °C for 3.5 h and then concentrated. The residue was diluted with dichloromethane and the organic phase was washed with saturated aqueous sodium bicarbonate and brine (2x), dried, and concentrated to a clear oil, which solidified on standing to leave **7** (1.25 g, 72%) as a bright white solid. ^{1}H NMR (400 MHz, chloroform-*d*) δ 8.96 (s, 1H), 7.53 (m, 2H), 6.88 (m, 2H), 4.20 (t, J = 5.9 Hz, 2H), 3.48 (t, J = 5.1 Hz, 4H), 2.86 (t, J = 5.8 Hz, 2H), 2.74 (m, 2H), 2.58 (t, J = 5.1 Hz, 4H), 1.59 (m, 2H), 1.46 (s, 9H), 0.98 (m, 3H). MS *m/z* 392.0 $(M+H)^+$.

***tert*-Butyl 4-(2-(4-(1-aminobutyl)phenoxy)ethyl)piperazine-1-carboxylate (8)**. Compound **7** (1.62 g, 4.14 mmol) in ethanol (50 mL) was warmed to promote dissolution and then cooled in an ice bath. To the stirred suspension were added 2N aqueous NaOH (20 mL) and 1:1 aluminum:nickel catalyst (3.3 g;

CAS 12635-27-7), in that order, with the catalyst added portion-wise over 30 min. The mixture was allowed to warm to room temperature and maintained there overnight. The suspension was filtered through celite with the pad washed copiously with ethyl acetate. The combined filtrate/washings were concentrated to leave a residue that was purified by column chromatography eluting with 95:5:0.5 dichloromethane: methanol: ammonia. Product fractions were concentrated to give **8** (1.45 g, 93%). ^{1}H NMR (400 MHz, chloroform-*d*) δ 8.95 (br s, 2H), 7.27 (d, *J* = 8.6 Hz, 2H), 6.87 (d, *J* = 8.5 Hz, 2H), 4.12 (m, 2 H), 3.99 (dd, *J* = 9.4, 5.6 Hz, 1H), 3.45 (t, *J* = 4.9 Hz, 4H), 2.82 (t, *J* = 5.5 Hz, 2H), 2.55 (t, *J* = 5.1 Hz, 4H), 1.70 (m, 2H), 1.47 (s, 9H), 1.21 (m, 2H), 0.85 (t, *J* = 7.3 Hz, 3H).

***tert*-Butyl 4-(2-(4-(1-(2-cyanoacetamido)butyl)phenoxy)ethyl)piperazine-1-carboxylate (9)**. An ice-cold solution of 2-cyanoacetic acid (0.214 g, 2.52 mmol) in DMF (7 mL) was treated sequentially with HOAt (CAS 39968-33-7; 0.343 g, 2.52 mmol)], HATU (CAS 148893-10-1; 0.957 g, 2.52 mmol)], diisopropylethylamine (1.32 ml, 7.55 mmol), and compound **8** (0.95 g, 2.52 mmol) dissolved in 5 mL of DMF. The resulting solution was stirred at room temperature for 42 h. The solution was diluted with ethyl acetate, washed with saturated aqueous sodium bicarbonate (2x) and brine, dried, and concentrated to leave a thick yellow oil that was purified by column chromatography, eluting first with 5% methanol in dichloromethane and then with 95:5:0.5 dichloromethane: methanol: ammonium hydroxide. Product fractions were concentrated to give **9** (0.99 g, 88%). ^{1}H NMR (400 MHz, chloroform-*d*) δ 7.16 (dd, *J* = 8.7, 2.1 Hz, 2H), 6.75 (dd, *J* = 8.5, 1.5 Hz, 2H), 5.90 (d, *J* = 8.2 Hz, 1H), 4.84 (p, *J* = 7.9 Hz, 1H), 4.07 (m, 2H), 3.64 (m, 1H), 3.45 (t, *J* = 5.0 Hz, 4H), 3.13 (q, *J* = 7.4 Hz, 1H), 2.80 (m, 2H), 2.52 (t, *J* = 5.0 Hz, 4H), 1.71 (m, 2H), 1.44 (s, 9H), 1.21 (m, 2H), 0.88 (td, *J* = 7.3, 1.2 Hz, 3H). MS *m/z* 445.2 $(M+H)^+$.

***tert*-Butyl(*E*)-4-(2-(4-(1-(2-cyano-3-(3,6-dichloropyridin-2-yl)acrylamido)butyl)phenoxy)ethyl)piperazine-1-carboxylate (10)**. A solution of compound **9** (0.72 g, 1.620 mmol) and 3,6-dichloropicolinaldehyde (1.14 g, 6.48 mmol) in ethanol (10 mL), 3-aminopropanoic acid (2.31 g, 25.9 mmol), and water (10 mL) was made up as described above for the synthesis of **5**. The mixture was stirred at room temperature for 19 h and then concentrated. The residue was diluted with ethyl acetate and the solution was washed with saturated aqueous sodium bicarbonate (2x), brine, dried, and concentrated to a solid that was purified by column chromatography eluting with 2:1 ethyl acetate:hexanes. Product fractions were concentrated to give **10** (0.18 g, 18%) as a white foam. ^{1}H NMR (400 MHz, chloroform-*d*) δ 8.61 (s, 1H), 7.74 (d, *J* = 8.5 Hz, 1H), 7.35 (d, *J* = 8.5 Hz, 1H), 7.24 (m, 2H), 6.89 (m, 2H), 6.78 (d, *J* = 8.0 Hz, 1H), 5.00 (q, *J* = 7.7 Hz, 1H), 4.10 (q, *J* = 6.5, 5.7 Hz, 2H), 3.45 (t, *J* = 5.0 Hz, 4H), 2.81 (t, *J* = 5.7 Hz, 2H), 2.52 (t, *J* = 4.8 Hz, 4H), 1.86 (m, 2H), 1.45 (s, 9 H), 1.26 (m, 2 H), 0.95 (t, *J* = 7.3 Hz, 4H). MS *m/z* 602.1 $(M+H)^+$.

(*E*)-2-Cyano-3-(3,6-dichloropyridin-2-yl)-*N*-(1-(4-(2-(4-(5-((3a*S*,4*S*,6a*R*)-2-oxohexahydro-1*H*-thieno[3,4-*d*]imidazol-4-yl)pentanoyl)piperazin-1-yl)ethoxy)phenyl)butyl)acrylamide (11; G9-biotin or DUB^{inh}-biotin). A stirred solution of compound **10** (0.153 g, 0.25 mmol) in dichloromethane (5 mL) was cooled in an ice bath and treated with trifluoroacetic acid (0.55 mL). The mixture was allowed to warm to room temperature over 2.5 h and then kept there for 1.5 h while being monitored for loss of starting material by TLC (10:90 methanol:dichloromethane). The solution was concentrated to

an oil that was pumped at high vacuum to leave a dark oil that was dissolved in DMF (5 mL) and cooled in an ice bath. (+)-Biotin *N*-hydroxysuccinimide ester (0.10 g, 0.29 mmol; synthesized by the method of Chan et al., *J. Amer. Chem. Soc.*, **2004**, 126 (44),14435–14446) and diisopropylethylamine (0.27 mL, 1.53 mmol) were added, and the mixture was stirred at 0 °C for 2 h and then at room temperature for 45 min. The mixture was partitioned between dichloromethane and water, and the organic phase was washed with water (2x) and brine, dried, and concentrated to an oil that was purified by column chromatography, eluting with 7% methanol in dichloromethane, to give **11** (68 mg, 32%) as a pale yellow oil. ^{1}H NMR (400 MHz, chloroform-*d*) δ 8.61 (s, 1H), 7.73 (d, *J* = 8.4 Hz, 1H), 7.45 (d, *J* = 8.5 Hz, 1H), 7.24 (m, 2H), 6.89 (m, 2H), 6.78 (m, 1H), 5.96 (br s, 1H), 5.42 (br s, 1H), 5.00 (q, *J* = 7.6 Hz, 1H), 4.51 (m, 1H), 4.30 (m, 1H), 4.10 (t, *J* = 5.5 Hz, 2H), 3.63 (d, *J* = 5.6 Hz, 2H), 3.48 (t, *J* = 5.2 Hz, 2H), 3.16 (td, *J* = 7.3, 4.5 Hz, 1H), 2.96-2.70 (m, 4H), 2.57 (dt, *J* = 18.2, 5.3 Hz, 4H), 2.35 (t, *J* = 7.4 Hz, 2H), 1.88-1.25 (m, 10 H), 0.95 (t, *J* = 7.3 Hz, 3H). HRMS *m/z*: calc., 728.2547; found, 728.2548 $(M+H)^{+}$. HPLC 96%.

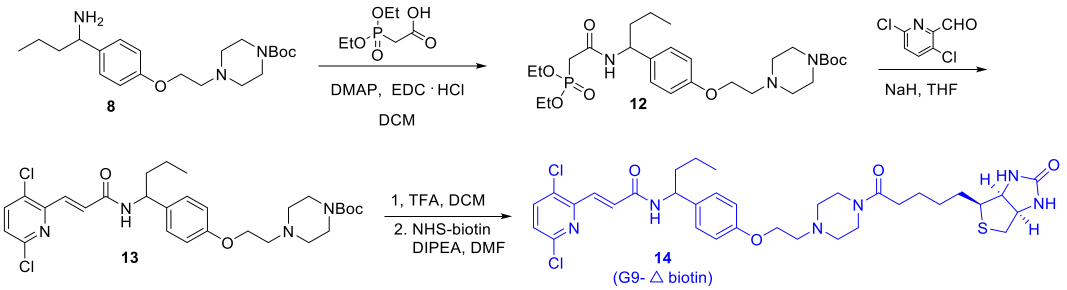

Synthetic scheme for compound G9Δ-biotin (ΔCN-biotin) (**14**)

***tert*-Butyl 4-(2-(4-(1-(2-(diethoxyphosphoryl)acetamido)butyl)phenoxy)ethyl)piperazine-1-carboxylate (12)**. To a solution of compound **8** (0.45 g, 1.19 mmol) and diethylphosphonoacetic acid (0.19 mL, 1.19 mmol) in DMF (8 mL) was added *N*-(3-dimethylaminopropyl)-*N'*-ethylcarbodiimide hydrochloride (EDC; 0.46 g, 2.38 mmol) and 4-dimethylaminopyridine (0.29 g, 2.38 mmol), and the resulting mixture was stirred at room temperature overnight. The solution was diluted with 0.5 N aqueous HCl (50 mL) and extracted with dichloromethane (3x). The combined extracts were washed with brine, dried, and concentrated *in vacuo* to leave **12** (0.46 g, 69%) as a white foam. ^{1}H NMR (400 MHz, chloroform-*d*) δ 7.35 (d, *J* = 8.2 Hz, 1H), 7.24 (dd, *J* = 8.4, 3.6 Hz, 2H), 6.76 (d, *J* = 8.2 Hz, 2H), 4.79 (q, *J* = 7.6 Hz, 1H), 4.42 (d, *J* = 4.2 Hz, 2H), 4.12-4.00 (m, 4H), 3.84 (br s, 4H), 3.49 (m, 4 H), 2.95 (m plus DMF), 1.76 (m, 2H), 1.40 (s, 9H), 1.25-1.15 (m, 8H), 0.91(t, *J* = 7.3 Hz, 3H). MS *m/z* 556.1 $(M+H)^{+}$.

***tert*-Butyl(*E*)-4-(2-(4-(1-(3-(3,6-dichloropyridin-2-yl)acrylamido)butyl)phenoxy)ethyl)piperazine-1-carboxylate (13)**. A suspension of sodium hydride (60% by weight; 0.1 g, 2.48 mmol) in THF (2 mL) was cooled in an ice bath and treated dropwise with a solution of compound **12** (0.46 g, 0.83 mmol) in dry THF (6 mL). The resulting suspension was stirred at room temperature for 45 min and then treated dropwise with a solution of 3,6-dichloropicolinaldehyde (0.146 g, 0.83 mmol) in THF (4 mL). The dark tan solution was stirred at room temperature for 2 h while being monitored by TLC (1:1 ethyl acetate:hexanes). The mixture was cautiously quenched with water and extracted with dichloromethane

(2x). The combined extracts were washed with brine, dried, and concentrated to a yellow oil that was purified by column chromatography eluting with 60:40 ethyl acetate:hexanes. The product fractions were concentrated and pumped *in vacuo* to give **13** (0.186 g, 39%) as a yellow foam. ^{1}H NMR (400 MHz, chloroform-*d*): δ 7.99 (dd, *J* = 14.9, 0.9 Hz, 1H), 7.63 (dd, *J* = 8.4, 0.8 Hz, 1H), 7.21 (t, *J* = 9.1 Hz, 3H), 7.08 (dd, *J* = 14.8, 0.8 Hz, 1H), 6.88 (d, *J* = 8.3 Hz, 2H), 5.99 (d, *J* = 8.2 Hz, 1H), 5.05 (t, *J* = 7.7 Hz, 1H), 4.10 (m, 2H), 3.45 (t, *J* = 5.0 Hz, 4H), 2.80 (t, *J* = 5.6 Hz, 2H), 2.54 (t, *J* = 5.2 Hz, 4H), 1.80 (m, 2H), 1.46 (s, 9H), 1.35 (m, 2H), 0.93 (m, 3H). MS *m/z* 578.0 $(M+H)^+$.

(*E*)-3-(3,6-Dichloropyridin-2-yl)-*N*-(1-(4-(2-(4-(5-((3a*S*,4*S*,6a*R*)-2-oxohexahydro-1*H*-thieno[3,4-*d*]imidazol-4-yl)pentanoyl)piperazin-1-yl)ethoxy)phenyl)butyl)acrylamide (14; G9Δ-biotin or ΔCN-biotin). A stirred solution of compound **13** (0.148 g, 0.26 mmol) in dichloromethane (5 mL) was cooled in an ice bath and treated dropwise with trifluoroacetic acid (0.8 mL). The resulting mixture was allowed to warm slowly to room temperature over 90 min while being monitored by TLC (10% methanol in dichloromethane). The solution was concentrated and pumped *in vacuo* to a constant weight. The residue was dissolved in DMF (5 mL) and the solution was cooled in an ice bath. Following treatment with (+)-biotin *N*-hydroxysuccinimide ester (0.101 g, 0.30 mmol) and diisopropylethylamine (0.27 mL, 1.54 mmol), the mixture was stirred at 0 °C for 90 min and then at room temperature for 30 min. The solution was diluted with ethyl acetate, washed successively with water (2x) and brine, dried, and concentrated to a yellow oil that was purified by column chromatography eluting with 8% methanol in dichloromethane. Product fractions were concentrated to leave a residue that was diluted with a small volume of 1:1 ethyl acetate:dichloromethane followed by filtration to remove turbidity. Concentration of the filtrate followed by pumping *in vacuo* to a constant weight left **14** (123 mg, 68%) as a white foam. ^{1}H NMR (400 MHz, chloroform-*d*) δ 7.94 (d, *J* = 14.9 Hz, 1H), 7.63 (d, *J* = 8.4 Hz, 1H), 7.22 (m, 3H), 7.11 (d, *J* = 14.9 Hz, 1H), 6.86 (d, *J* = 8.6 Hz, 2H), 6.23 (d, *J* = 8.1 Hz, 1H), 5.66 (br s, 1H), 5.07 (t, *J* = 7.6 Hz, 2H), 4.47 (m, 1H), 4.28 (m, 1H), 4.13 (t, *J* = 5.3 Hz, 2H), 3.65 (m, 2H), 3.53 (m, 2H), 3.15 (q, *J* = 6.7 Hz, 1H), 2.90 (dd, *J* = 12.7, 4.9 Hz, 3H), 2.78 - 2.70 (m, 5H), 2.37 (t, *J* = 7.4 Hz, 2H), 1.70 - 1.44 (m, 10 H), 0.95 (t, *J* = 7.3 Hz, 3H). HRMS *m/z*: calc., 703.2595; found, 703.2592 $(M+H)^+$. HPLC 96%.
